# SMS nudges as a tool to reduce tuberculosis treatment delay and pretreatment loss to follow-up. A randomized controlled trial

**DOI:** 10.1371/journal.pone.0218527

**Published:** 2019-06-20

**Authors:** Adam Wagstaff, Eddy van Doorslaer, Ronelle Burger

**Affiliations:** 1 Development Research Group, World Bank, Washington, DC, United States of America; 2 Erasmus School of Health Policy and Management and Erasmus School of Economics, Erasmus University Rotterdam, Rotterdam, The Netherlands; 3 Department of Economics, Stellenbosch University, Stellenbosch, South Africa; University of KwaZulu-Natal, SOUTH AFRICA

## Abstract

**Background:**

TB persists despite being relatively easy to detect and cure because the journey from the onset of symptoms to cure involves a series of steps, with patients being lost to follow-up at each stage and delays occurring among patients not lost to follow-up. One cause of drop-off and delay occurs when patients delay or avoid returning to clinic to get their test results and start treatment.

**Methods:**

We fielded two SMS interventions in three Cape Town clinics to see their effects on whether people returned to clinic, and how quickly. One was a simple reminder; the other aimed to overcome “optimism bias” by reminding people TB is curable and many millions die unnecessarily from it. Recruits were randomly assigned at the clinic level to a control group or one of the two SMS groups (1:2:2). In addition to estimating effects on the full sample, we also estimated effects on HIV-positive patients.

**Results:**

SMS recipients were more likely to return to clinic in the requested two days than the control group. The effect was smaller in the intent-to-treat analysis (52/101 or 51.5% vs. 251/405 or 62.0%, p = 0.05) than in the per-protocol analysis (50/97 or 51.5% vs. 204/318 or 64.2%, p = 0.03). The effect was larger among HIV-positives (10/35 or 28.6% vs. 97/149 or 65.1%, p<0.01). The effects of SMS messages diminished as the interval increased: significant effects at the 5% level were found at five and 10 days only among HIV-positives. The second SMS message had larger effects, albeit not significantly larger, likely due in part to lack of statistical power.

**Conclusions:**

At 2 U.S. cents per message, SMS reminders are an inexpensive option to encourage TB testers to return to clinic, especially when worded to counter optimism bias.

## Introduction

An estimated 1.6 million people died from tuberculosis (TB) globally in 2016 [1]. South Africa (the setting for the present study) has one of the world’s highest mortality rates and the highest incidence rate [1]. TB persists despite being relatively easy to detect and cure[1], the reason being that the journey from the onset of symptoms to cure involves a series of steps, with patients being lost to follow-up (LTFU) at each stage and delays occurring among patients who are not lost to follow-up [2–4]. Some people with TB symptoms never contact a provider, and those who do often delay seeking care (‘patient delay’) [3]. Not every contact with a provider leads to a diagnosis, and, when it does, there is often another delay (‘diagnostic delay’) [3]. Not every diagnosis results in appropriate treatment, and, even when it does, there can be another delay (‘treatment delay’) [3]. Finally, not everyone who starts treatment successfully completes it (‘cure delay’) [3]. In South Africa, the net effect of these drop-offs in the treatment ‘cascade’ is that only an estimated 53% of persons with TB complete treatment [4]. It is estimated that pre-treatment or initial LTFU in South Africa is 11–25% [4–8] and accounts for as much as 25% of that country’s drop-off [4]. Reducing treatment delay and initial LTFU are therefore potentially important elements in any strategy to reduce TB mortality in South Africa.

The question arises whether short-message service (SMS) messages might increase the share of (and speed with which) patients who have tested for TB return to the clinic to get their test results and start appropriate treatment. In South Africa, where initial diagnosis is increasingly done using GeneXpert [9], government guidelines [10] urge clinics to give patients a return date within two days on which they get their test results and start appropriate treatment. If a patient does not return on the specified date for their results, and the results confirm drug-susceptible or drug-resistant TB, the patient should be traced by the clinic, and started on treatment “as soon as possible”. The guidelines acknowledge the added complexities in diagnosing TB in HIV-positive patients but emphasize the need to start them on treatment as quickly as possible, given the inferior TB treatment outcomes of this group, reflecting the increased risk of rapid progression and the host’s impaired ability to contain TB infection [11–14].

The effects of text messages and other digital technologies have been studied in a variety of contexts relating to TB treatment adherence and completion, although the quality of the studies is not, in general, considered high [15–17]. However, no study to date has examined the effects of text messages and other digital technologies on the initiation of treatment during the diagnosis stage. A review by the Collaborative Group on the Impact of Digital Technologies on TB [16] concludes that “It will be relevant to explore the possible influence of digital technologies on behavior change at other points on the patient pathway–for example, in preventing initial loss-to-follow-up before TB treatment initiation” (p.9).

Behavioral economics suggests the content of the SMS message may affect its effect on how quickly (and if at all) a TB tester returns to collect their results. The most basic message is a simple appointment reminder. These have worked in multiple settings, including encouraging patients to keep appointments [18]. One possible explanation–particularly relevant for poor populations, where TB is commonest–is that people tend to prioritize tasks that meet today’s needs (e.g. getting children to and from school, turning up to work, ensuring the family has enough food for dinner, etc.) over those that meet longer-term needs (e.g. returning to clinic to get a TB test result), especially if in the latter tasks there is greater uncertainty over the benefits involved [19]. A more sophisticated message would contain (in addition to the simple reminder) text that tries to counter optimism bias–a tendency to overestimate the likelihood of favorable future outcomes and underestimate the likelihood of unfavorable future outcomes, such as having TB [20]. This more complex message is also motivated by research showing that patient knowledge and beliefs are among the factors that apparently contribute to pre-treatment LTFU and delay [3, 5, 21, 22].

Our paper reports the result of a trial testing two different SMS reminders aimed at increasing the share of (and speed with which) patients who have contacted a provider–and have been tested–return to the clinic to get their test results and start appropriate treatment.

## Materials and methods

### Trial design and participants

This RCT was undertaken at three clinics in the Cape Town area selected from a shortlist of the highest burden clinics in the Cape Town area, eliminating those that were already involved in another trial that might have contaminated our results. The clinics were also highly receptive to our request to participate in the study.

Patients in all three clinics have a dedicated TB waiting room to which patients already being treated are directed along with patients who are considered possible TB cases. We aimed to recruit 90% of patients over the age of 18 in the three waiting rooms during the study period. We asked all those not already being treated for TB if they were willing to participate in the study, and if so to complete a questionnaire on a tablet prior to being evaluated by the TB nurses and producing their sputum sample. Patients agreeing to completing the questionnaire were randomly assigned to a control group or one of two intervention groups, with an allocation ratio of 1:2:2. Recruitment was done by the fieldworkers in each of the three clinics. The patient’s information was recorded via a tablet in the clinic, and this information was then uploaded to a central database that was inaccessible to the fieldworkers.

### Ethical approval, protocol, trial registration and checklists

The study was approved by the Stellenbosch University Ethics Committee on November 8, 2016 and subsequently extended (reference number SU-HSD-003565 “A Behavioral and Economic Analysis of TB Non-Initiation in Cape Town, South Africa”). The IRB approval is available as supporting information as S1 File. The protocol is available as S2 File, and the SPIRIT and CONSORT checklists are available as S3 File and S4 File.

Due to an oversight, the trial was registered only retrospectively with the Pan African Clinical Trial Registry on December 14, 2018 (reference number PACTR201812631160518 https://pactr.samrc.ac.za/TrialDisplay.aspx?TrialID=5780). Despite retrospective registration, we closely followed the protocol outlined in our IRB application. Recruitment was from 2 October 2017 until 15 December 2017. Fieldworkers continued visiting clinics and phoning patients until mid-February 2018 to collect data on patients’ return-to-clinic date, test results and treatment start date.

### Interventions

The control group received no SMS reminder message. The first intervention group (SMS1) received a simple SMS reminder to return to the clinic to collect their TB test results. Clinic staff told patients to return in two (working) days’ time to get their results, and the SMS was sent via a bulk SMS service around 6-7pm the evening before. The message in English read “Don't forget to collect your results from the clinic tomorrow.” The second intervention group (SMS2) received a longer SMS message reminding them to return to the clinic, and that people die unnecessarily from TB because it can be cured. The message in English read: “Don't forget to collect your results from the clinic tomorrow. 96,000 South Africans die every year from TB. This should not be–TB can be cured”. Both messages were translated into and sent in Xhosa for those who had said in their baseline interview that this was their native language.

We report two sets of results: one where we combined the two intervention groups and tested whether outcomes were affected by the individual receiving any SMS; and a second where we separated the two interventions groups to allow us to test whether the two SMSs had different effects.

We examined the records of the bulk SMS service to see if the delivery of the SMS message failed, or if the wrong SMS was sent, or if patients in the control group ended up receiving an SMS message. In all these cases, the intended “treatment” was not delivered. Since TB is a communicable disease and phone-sharing is common in South Africa, we also checked whether patients were sharing a phone with someone in our study. In cases where phone-sharing occurred across intervention groups, we could not be sure that the intended “treatment” was delivered to either party. In our main analysis, we kept all cases, irrespective of whether we know or think the correct treatment may not have been delivered–we label this analysis an “intent-to-treat” (ITT) analysis. We also report the results of a second analysis that excludes such cases–we label this a “per protocol” (PP) analysis.

### Outcomes

Our outcomes captured whether people return for their tests and how quickly. We report the percentage of recruits returning at all, the median days to return, and the percentages returning within two business days (the specified date to collect test results), five business days (one week), 10 business days (two weeks), and 20 business days (four weeks), 10 and 20 days being two commonly-used cut-offs in estimating pre-treatment LTFU [5, 7, 23]. We also plot “survival” curves for both groups showing the time to return-to-clinic. Data on return-to-clinic date were obtained via fieldworker interactions with returning patients in the TB waiting room, conversations with clinic staff, and where necessary, phone follow-up conversations with patients and inspection of clinic records. The time-to-return variable (measured in days) was adjusted for weekends and public holidays when the clinics in question were closed.

For all outcomes, we report results for the full sample and the subsample who are HIV-positive. We obtained data on HIV status (if known) from two sources: Cape Town’s Patient Record and Health Management Information System (PREHMIS), which also includes patients not on TB treatment, and South Africa’s Electronic Tuberculosis Register (ETR.Net). We first did a fuzzy (bigram) matching against ETR.net records by name [24]. We classified a match as sufficiently close if the (Jaccard) similarity score was at least 0.8 (similarity scores above 0.8 but less than 1.0 were typically due to misspellings or middle names being omitted). In these close matches, we took the patient’s HIV status data from ETR.Net. In other cases, we looked for a close enough match in the PREHMIS data. Here we used a two-stage matching process: first, we did an exact matching by patient number; then we did a fuzzy (bigram) matching by name. In each case, we assessed the quality of the name match by computing a (Jaccard) similarity score. We used whichever method gave the higher score (often the matched name was the same), and again we classified a match as sufficiently close if the (Jaccard) similarity score was at least 0.8. For 82% of our sample, we were able to find HIV status from either ETR.Net or PREHMIS: 35% of our sample was known to be HIV-positive.

### Sample size

We expected to recruit around 80 patients per week across the three clinics and planned on recruiting over a nine-week period beginning October 3, 2017, anticipating 720 recruits in total. In the event, recruitment was slower, averaging just 60 patients per week. We therefore continued recruiting until the start of the Christmas holiday, recruiting and randomizing 649 patients, who we split across the control group and two intervention groups in the ratio 1:2:2.

There were essentially no baseline data for us to use to assess power ex ante. The only data point we had was an estimate of the fraction of TB-positives who do not begin treatment, which has been estimated to be as high as 25% [4]. Assuming this applies equally to TB-negatives, and therefore only 75% of testers return at any stage to get their results, a sample size of 649 is large enough to detect, with 83% power, a 10 percentage-point ITT effect on returning at any stage, assuming a significance level of 5%.

### Randomization

Fieldworkers recruited patients as they came into each clinic, entering the data from the patient interview on their tablet, with cases ordered by recruitment date and time. The data were uploaded at the end of each day to a central database and recruits were assigned that evening to one of the three groups by the study coordinator. The process involved the study coordinator working down three clinic-specific randomization sequences generated previously by the authors using a web-based randomization tool [25], a clinic-specific seed and block sizes of 10. The first recruit on Day 1 from Clinic A was assigned to whatever group was indicated in the first row of Clinic A’s randomization sequence, the second to the group indicated in the second row, and so on until all recruits that day from Clinic A had been assigned. If, say, six patients were recruited on Day 1 in Clinic A, the first recruit on Day 2 would be assigned to the group indicated on the seventh row of the randomization sequence, and so on. In the randomization process, we stratified by clinic to ensure we achieved the desired proportions (and a high degree of balance) across the three groups within each clinic. The randomized sequences were concealed from fieldworkers who could not therefore anticipate the allocation of patients being recruited or know the allocation of patients after the fact. The study depended, of course, on some patients receiving an SMS, so blinding of patients to their “treatment” status was neither feasible nor desirable. Clinic staff, like fieldworkers, were not told of the assignments.

### Statistical methods

For the outcomes that are simple proportions–the fraction of recruits returning at all and the fraction returning within a specific number of days–we computed Pearson’s χ^2^ test of equal proportions. For the days-to-return outcome, we computed a t-test of equal means. Estimation was done using Stata 15.0.

## Results

### Recruitment and follow-up

Recruitment was from 2 October 2017 until 15 December 2017. Fieldworkers continued visiting clinics and phoning patients until mid-February 2018 to collect data on patients’ return-to-clinic date, test results and treatment start date. A two-month pre-experimental phase preceded the start of the randomized trial. During this period, patients were simply interviewed and did not receive any intervention. The recruitment and interview processes were developed and fine-tuned, the fieldworkers became familiar with their tasks, gained the trust of the clinic staff and became a part of the clinic workflow, and the study team developed methods for reconciling data from our baseline interview and the clinic records.

### Participant flow and numbers analyzed

**Fig 1:**
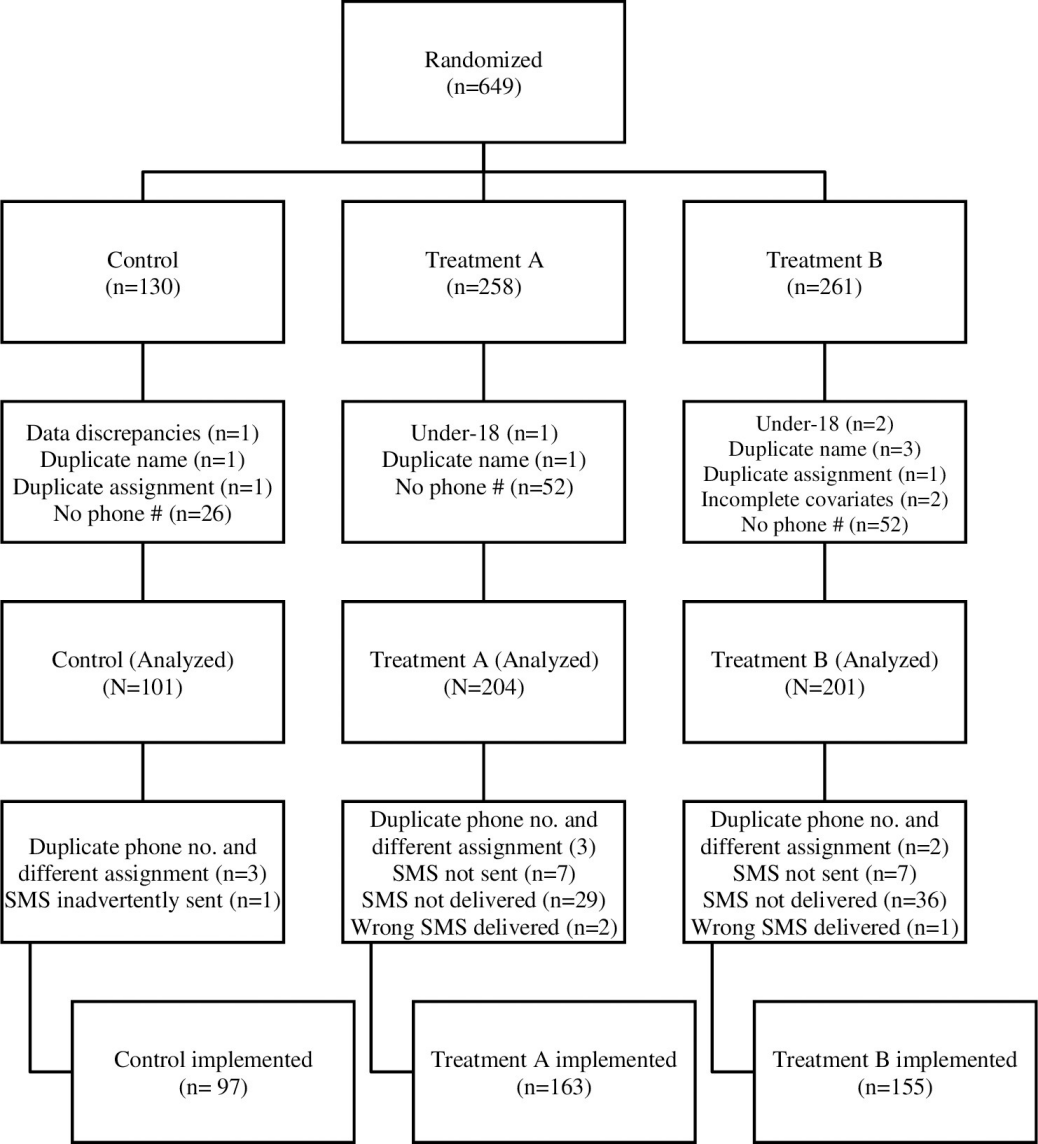
Flow chart of participants.

Fig 1 shows the participant flow. A total of 649 cases were randomized across the three arms. Of these, 506 were retained for the ITT analysis; the reasons for excluding the 143 cases are shown in Fig 1 by treatment arm. Most exclusions were because the patient did not provide a (valid) mobile phone number for follow-up (the phone could have been of a family member, whether or not living in the household). Of the 506 ITT cases, 415 were retained for the PP analysis; the reasons for excluding the 91 cases are also shown in Fig 1 by treatment arm. Most exclusions in this case were due to SMS delivery failure. S5 File shows the distribution of the sample across the three clinics and intervention groups.

### Baseline data

Table 1 and S6 File show for the full sample and PP subsample baseline differences between the control group and the intervention groups in the likely influences on return-to-clinic and treatment initiation. The baseline characteristics were well balanced across the groups.

**Table 1:** Baseline characteristics (full sample).

	Control (n = 101)		SMS (n = 405)		SMS1 (n = 204)		SMS2 (n = 201)	
Variable	N/mean	%/SD	N/mean	%/SD	N/mean	%/SD	N/mean	%/SD
Female	42	41.6	183	45.2	84	41.2	99	49.3
College educated	14	13.9	59	14.6	34	16.7	25	12.4
Employed	63	62.4	249	61.5	130	63.7	119	59.2
Electricity	96	95.0	378	93.3	187	91.7	191	95.0
Running water	12	11.9	30	7.4	17	8.3	13	6.5
TV	88	87.1	359	88.6	181	88.7	178	88.6
Fridge	84	83.2	346	85.4	168	82.4	178	88.6
Satellite	38	37.6	130	32.1	73	35.8	57	28.4
Car	20	19.8	60	14.8	35	17.2	25	12.4
Mobile phone in household	92	91.1	356	87.9	180	88.2	176	87.6
Hungry	27	26.7	127	31.4	63	30.9	64	31.8
Age	39.89	12.80	39.87	11.95	39.14	11.48	40.61	12.40

### Outcomes and estimation

**Fig 2:**
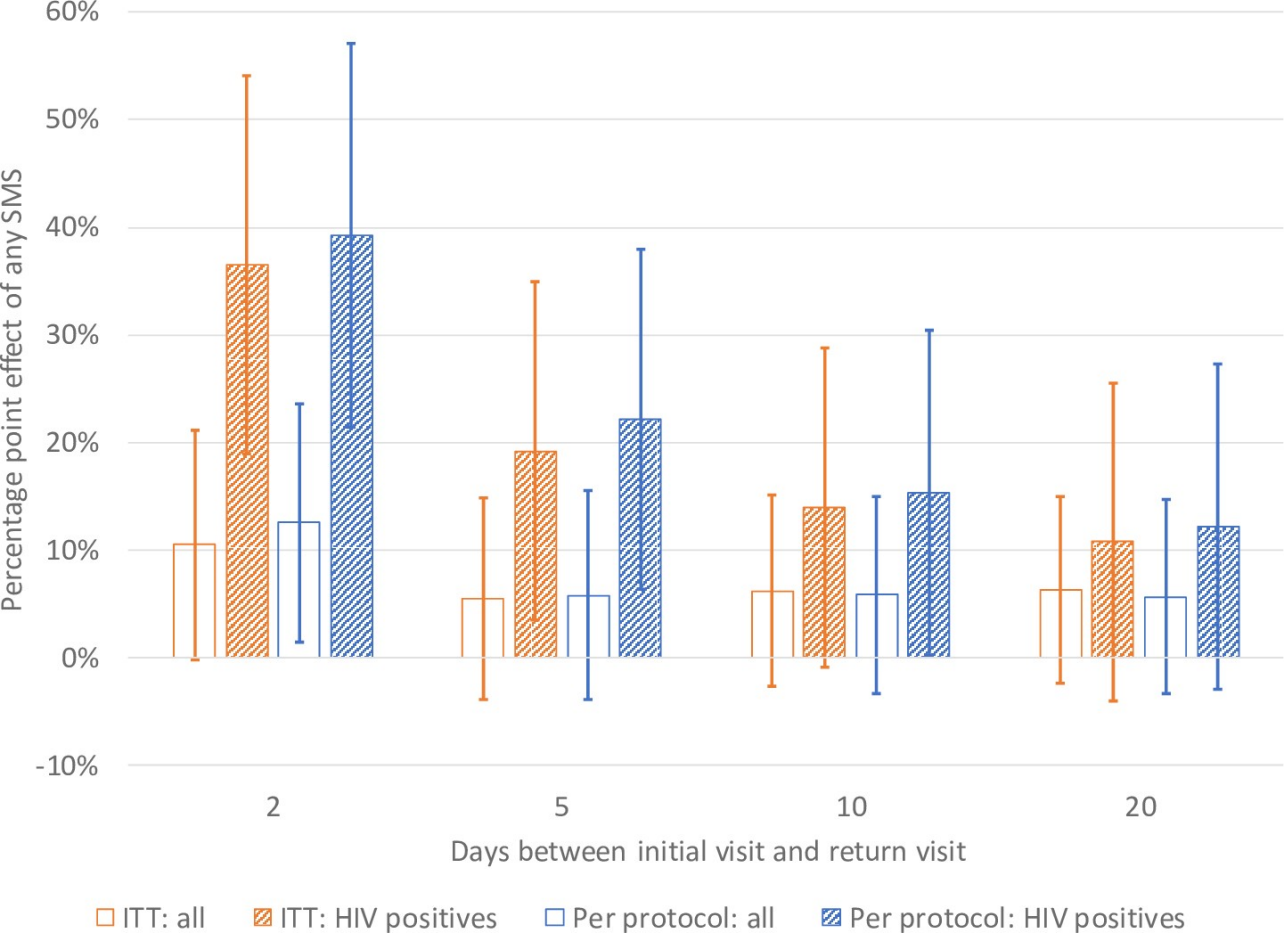
ITT and per-protocol results: Percentage point effect from any SMS. Lines represent 95% confidence intervals.

**Fig 3:**
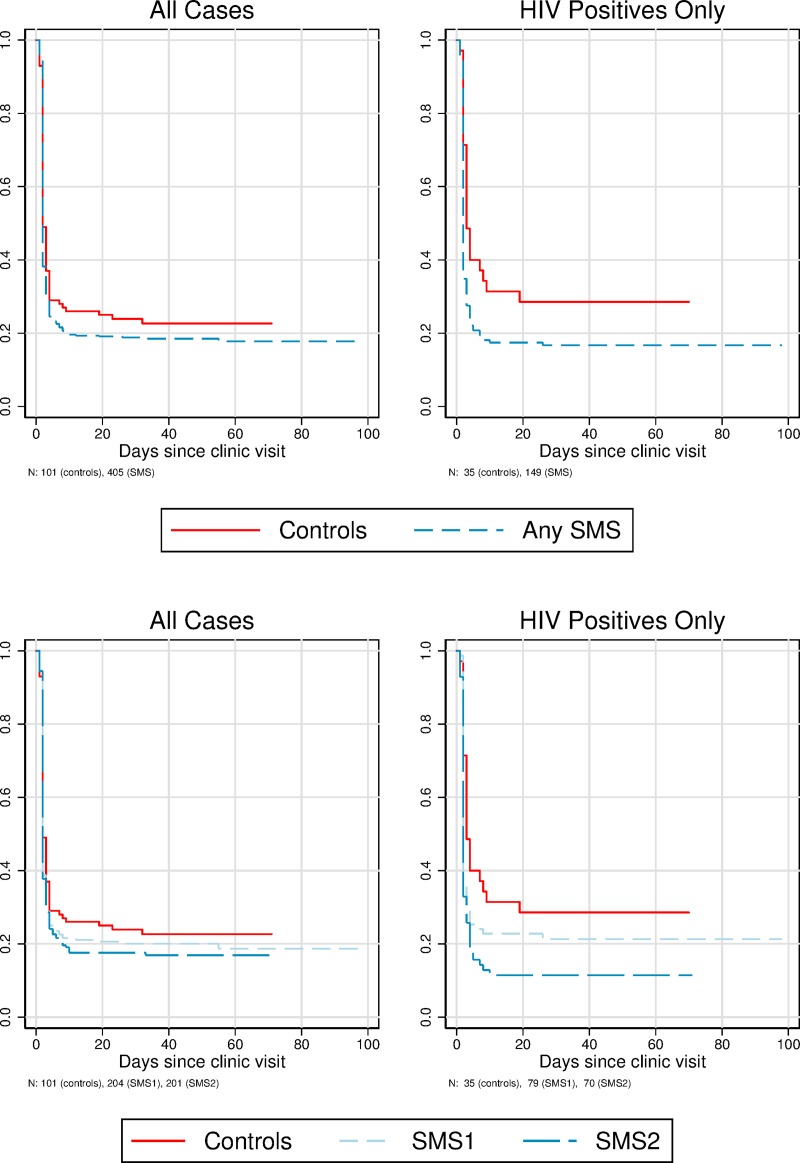
Survival curves for non-return to clinic (ITT sample). Top panel shows combined treatment groups (any SMS). Bottom panel shows separate treatment groups.

The ITT effects are, unsurprisingly, somewhat smaller than the PP effects (Tables 2 and 3, and Fig 2). For example, in the ITT analysis, 62.0% of patients receiving an SMS message returned to clinic in two days compared to 51.5% in the control group (p = 0.05) (a 10.5 percentage point effect; 95% confidence interval -0.34 to 21.32), while in the PP analysis, 64.2% of patients receiving an SMS message returned to clinic in two days compared to 51.5% in the control group (p = 0.03) (a 12.7 percentage point effect; 95% confidence interval 1.35 to 23.86). The ITT “survival” curves in Fig 3, which plot the cumulative fraction not returning at different delays since the initial visit, show a clear PP effect of SMS messages. The largest effects of SMS messages are at two days after the initial visit–the appointed time to return and collect test results; the effect is smaller thereafter (Tables 2 and 3, and Fig 2).

**Table 2:** Intent-to-treat results.

	N	Controls	Any SMS	p	SMS1	SMS2	p1	p2	p3
**Returned (as % cases analyzed)**									
Full sample	506	78/101 (77.2%)	331/405 (81.7%)	0.304	164/204 (80.4%)	167/201 (83.1%)	0.521	0.220	0.483
HIV positive	184	25/35 (71.4%)	124/149 (83.2%)	0.110	62/79 (78.5%)	62/70 (88.6%)	0.414	0.028	0.100
**Time-to-return (mean days)**									
Full sample	506	12.9	10.6	0.260	11.5	9.7	0.552	0.140	0.307
HIV positive	184	16.4	10.5	0.115	12.4	8.4	0.355	0.044	0.203
**Returned within 2 business days (as % recruits)**									
Full sample	506	52/101 (51.5%)	251/405 (62%)	0.054	125/204 (61.3%)	126/201 (62.7%)	0.103	0.062	0.770
HIV positive	184	10/35 (28.6%)	97/149 (65.1%)	0.000	50/79 (63.3%)	47/70 (67.1%)	0.001	0.000	0.623
**Returned within 5 business days (as % recruits)**									
Full sample	506	72/101 (71.3%)	311/405 (76.8%)	0.249	155/204 (76%)	156/201 (77.6%)	0.377	0.228	0.697
HIV positive	184	21/35 (60%)	118/149 (79.2%)	0.017	59/79 (74.7%)	59/70 (84.3%)	0.114	0.006	0.150
**Returned within 10 business days (as % recruits)**									
Full sample	506	75/101 (74.3%)	326/405 (80.5%)	0.167	160/204 (78.4%)	166/201 (82.6%)	0.415	0.089	0.291
HIV positive	184	24/35 (68.6%)	123/149 (82.6%)	0.063	61/79 (77.2%)	62/70 (88.6%)	0.328	0.012	0.068
**Returned within 20 business days (as % recruits)**									
Full sample	506	76/101 (75.2%)	327/405 (81.5%)	0.155	159/204 (79.1%)	168/201 (84%)	0.447	0.067	0.206
HIV positive	184	25/35 (71.4%)	120/149 (82.2%)	0.152	58/79 (76.3%)	62/70 (88.6%)	0.582	0.028	0.053

Notes: p for returned (as % recruits) and returned within *x* business days (as % recruits) is Pearson’s χ^2^ test of equal proportions (of returners / returners-within-*x*-days) across intervention and control groups. p for time-to-return (mean days) is a test of equality of means. p_1_ is test for SMS1 vs. control, p_2_ is test for SMS2 vs. control, p_3_ is test of equality across the two SMS groups.

**Table 3:** Per-protocol results.

	N	Controls	Any SMS	p	SMS1	SMS2	p1	p2	p3
**Returned (as % cases analyzed)**									
Full sample	415	75/97 (77.3%)	262/318 (82.4%)	0.263	132/163 (81%)	130/155 (83.9%)	0.478	0.194	0.499
HIV positive	147	25/35 (71.4%)	95/112 (84.8%)	0.074	48/60 (80%)	47/52 (90.4%)	0.339	0.022	0.127
**Time-to-return (mean days)**									
Full sample	415	12.9	10.3	0.225	11.1	9.4	0.480	0.142	0.382
HIV positive	147	16.4	9.4	0.055	11.3	7.2	0.231	0.028	0.220
**Returned within 2 business days (as % recruits)**									
Full sample	415	50/97 (51.5%)	204/318 (64.2%)	0.026	104/163 (63.8%)	100/155 (64.5%)	0.052	0.041	0.895
HIV positive	147	10/35 (28.6%)	76/112 (67.9%)	0.000	39/60 (65%)	37/52 (71.2%)	0.001	0.000	0.487
**Returned within 5 business days (as % recruits)**									
Full sample	415	70/97 (72.2%)	248/318 (78%)	0.236	126/163 (77.3%)	122/155 (78.7%)	0.353	0.235	0.762
HIV positive	147	21/35 (60%)	92/112 (82.1%)	0.007	46/60 (76.7%)	46/52 (88.5%)	0.086	0.002	0.104
**Returned within 10 business days (as % recruits)**									
Full sample	415	73/97 (75.3%)	258/318 (81.1%)	0.208	129/163 (79.1%)	129/155 (83.2%)	0.467	0.123	0.352
HIV positive	147	24/35 (68.6%)	94/112 (83.9%)	0.046	47/60 (78.3%)	47/52 (90.4%)	0.291	0.010	0.083
**Returned within 20 business days (as % recruits)**									
Full sample	415	74/97 (76.3%)	259/318 (82%)	0.216	128/163 (79.5%)	131/155 (84.5%)	0.544	0.103	0.247
HIV positive	147	25/35 (71.4%)	92/112 (83.6%)	0.111	45/60 (77.6%)	47/52 (90.4%)	0.505	0.022	0.070

Notes: p for returned (as % recruits) and returned within *x* business days (as % recruits) is Pearson’s χ^2^ test of equal proportions (of returners / returners-within-*x*-days) across intervention and control groups. p for time-to-return (mean days) is a test of equality of means. p_1_ is test for SMS1 vs. control, p_2_ is test for SMS2 vs. control, p_3_ is test of equality across the two SMS groups.

We found larger effects among HIV-positive patients (Tables 2 and 3, and Fig 2). For example, in the ITT analysis, 65.1% of HIV-positive patients receiving an SMS message returned to clinic in two days compared to 28.6% of HIV-positive patients in the control group (p<0.01). We found significant effects at the 5% level among HIV-positive patients also at five days in both the ITT and PP results, and at 10 days in the PP results.

We also found larger effects for the second SMS message in both the ITT and PP results (Tables 2 and 3, Fig 3 and S7 File). The gap between the effects of the two messages was larger among HIV-positive patients. In this group, we found significant effects (at the 5% level) of the second SMS on all six outcomes in both the ITT and PPP analyses. By contrast, among the those who were not known to be HIV-positive, we did not find significant effects on any outcome (S8 File). Across the four outcomes with a specific duration (return within two days, return within one week, etc.), the average gap between the two SMS effects is 10 percentage points among HIV-positive patients (similar to the gap for the ever-returned outcome) and -1 percentage point among the rest of the sample. None of the differences in effects between the two SMS messages are significant at the 5% level, likely reflecting a lack of statistical power.

We explored differences across clinics in the ITT analysis. The smallest effect (2.2 percentage points; 95% confidence interval -13.96 to 18.26) was in Clinic A where 57.4% (100/174) of patients receiving an SMS message returned to clinic in two days compared to 55.3% (21/26) in the control group (p = 0.79). The next largest effect (10.7 percentage points; 95% confidence interval -13.56 to 35.00) was in Clinic B where 71.8% (51/71) of patients receiving an SMS message returned to clinic in two days compared to 61.1% (11/18) in the control group (p = 0.38). The largest effect (20.8 percentage points; 95% confidence interval 3.07 to 38.60) was in Clinic C where 62.5% (100/160) of patients receiving an SMS message returned to clinic in two days compared to 41.7% (15/36) in the control group (p = 0.02). These differences are not statistically significant: the p-value for the 8.6 percentage point difference between Clinic A and Clinic B is 0.57; the p-value for the 10.1 percentage point difference between Clinic B and Clinic C is 0.52; and the p-value for the 18.7 percentage point difference between Clinic A and Clinic C is 0.12. At least in some of these cases, statistical power is likely to be the issue.

## Discussion

### Limitations

We did not follow up patients over the course of the TB treatment (if any) and therefore cannot provide any estimate of the health gain associated with quicker clinic return. Our sample size was likely too small to detect statistically significant differences in effects between the two SMS messages and across clinics.

### Generalizability

The findings of our trial in three high-TB patient volume clinics in the Greater Cape Town area do not necessarily generalize to clinics elsewhere in this part of South Africa let alone other parts of the country or other countries. The differences in effects across clinics are appreciable and their lack of statistical significance is likely due in part to lack of statistical power. This suggests caution in generalizing from these three clinics and raises the question of why the effects vary across them–are there clinic-specific reasons or do the differences in effects reflect differences in the communities served by the clinics? The gap between our ITT and PP results also shed some light on the issue of generalizability. The gap between our ITT and PP results reflects the extent of phone-sharing and swapping SIM cards between phones, cell phone provider changes, the challenge of charging phones, and the reliability of the methods used to send SMS messages. In our study, cell-phone sharing was fairly limited among study participants. More important was the fact that, in our study, around 20% of those with a phone did not receive the (intended) SMS message, either because of human error in queuing the message (5%), or non-delivery of the message (15%). Increased automation and longer-life batteries would likely increase the fraction of intended SMS messages getting delivered, which would push the ITT effects closer to the PP effects.

### What our study adds to existing evidence

Three literature reviews on the subject of the effects of SMS messages on TB treatment and adherence [15–17] all concluded that there was little evidence of significant benefits, either because the studies were flawed or because no effects were found. Only one large recent RCT, conducted in Anhui province in China [26] found significantly higher treatment completion rates and lower treatment interruption and missed doses rates in the SMS than the control group. However, this intervention is not comparable to our study as it examined the effect of very intensive daily one-way SMS reminders to improve adherence rather than the one-shot SMS reminder to promote initiation that we evaluated. We therefore appear to be the first to document a relevant effect of SMS reminders on TB treatment initiation. As such, our study responds to the recent concern that “evidence regarding the impact of digital adherence technologies on TB outcomes remains limited” [27].

### Interpretation

Our results suggest that one SMS message successfully transmitted on the evening before the due clinic return date substantially increases the likelihood of a patient coming back to get their test results on the scheduled day–in the PP analysis, by 13 percentage points among the sample as a whole and by 39 percentage points among HIV-positives. They have a smaller effect on the likelihood of returning at longer intervals. The smaller effects for longer intervals are consistent with the possibility of clinics contacting non-returnees testing positive starting the day after they were supposed to return, per the health department guidelines, and also with many patients coming back of their own accord, just not on the scheduled day. With a policy in place to contact non-returning TB positives after the scheduled return date has passed, the SMS may be less useful at combatting pre-treatment LTFU, and more useful at helping clinics get people back on the requested day, thereby avoiding the need to trace them and contact them if they fail to return.

Whether increases in two-day rates-of-return of 13 and 39 percentage points for the full sample and for HIV-positives respectively are economically significant depends on the costs and benefits involved. SMS message reminders are easy to automate and very cheap to deliver: assuming a functional administrative data and communication system with reliable and up-to-date phone numbers and language preferences, the incremental cost of dispatching messages is just 2 U.S. cents. Given the negative effects of treatment delay on the health of the patient [28–30], and the infection risk that treatment delay poses to others, the benefits of these percentage point increases in the likelihood of the patient returning on the scheduled day to get their results seems likely to exceed 2 U.S. cents.

## Supporting information

S1 File IRB approval. (PDF)

S2 File IRB protocol. (PDF)

S3 File SPIRIT checklist. SPIRIT 2013 Checklist: Recommended items to address in a clinical trial protocol.
(DOC)

S4 File CONSORT checklist. CONSORT 2010 checklist of information to include when reporting a randomized trial.
(DOC)

S5 File Table Distribution of sample across three study clinics. (DOCX)

S6 File Table Baseline characteristics (per-protocol sample). (DOCX)

S7 File Fig SMS1 and SMS2 results (per protocol). (DOCX)

S8 File Table Results for subsample not known to be HIV-positive. (DOCX)
