# Supplementary material for: SMS nudges as a tool to reduce tuberculosis treatment delay and pretreatment loss to follow-up. A randomized controlled trial
Source: PLoS One. 2019 Jun 20;14(6):e0218527. doi: 10.1371/journal.pone.0218527 (PMC6586322; doi:10.1371/journal.pone.0218527)
Supplement: S1 File — (PDF) [file pone.0218527.s001.pdf]

S1 Text. IRB Approval

UNIVERSITEIT·STELLENBOSCH·UNIVERSITY
jou kennisvennoot • your knowledge partner

Approved with Stipulations

Response to Modifications- (New Application)

08-Nov-2016
Jacobs, Rochelle R

Proposal #: SU-HSD-003565
Title: A Behavioral and Economic Analysis of TB Non-Initiation in Cape Town, South Africa

Dear Miss Rochelle Jacobs,

Your **Response to Modifications** - ***(New Application)*** received on **19-Oct-2016**, was reviewed by members of the **Research Ethics Committee: Human Research (Humanities)** via Expedited review procedures on **07-Nov-2016**.

Please note the following information about your approved research proposal:

Proposal Approval Period: **08-Nov-2016 -07-Nov-2017**

The following stipulations are relevant to the approval of your project and must be adhered to:
1) It is unclear how privacy will be ensured "through the use of tablets"? The REC still recommends that the participants be afforded the opportunity to complete the survey in private (even if this is just in a corner of the waiting room) should they have questions.
2) Informed consent form (ICF) Patient journeys – The researcher is requested to indicate on this document where the interview will take place. It should be at a time and place convenient for the participant. Will the interviews be recorded? If so, explicit permission should be sought via this ICF.

Please provide a letter of response to all the points raised IN ADDITION to HIGHLIGHTING or using the TRACK CHANGES function to indicate ALL the corrections/amendments of ALL DOCUMENTS clearly in order to allow rapid scrutiny and appraisal.

Please take note of the general Investigator Responsibilities attached to this letter. You may commence with your research after complying fully with these guidelines.

Please remember to use your **proposal number** (**SU-HSD-003565**) on any documents or correspondence with the REC concerning your research proposal.

Please note that the REC has the prerogative and authority to ask further questions, seek additional information, require further modifications, or monitor the conduct of your research and the consent process.

Also note that a progress report should be submitted to the Committee before the approval period has expired if a continuation is required. The Committee will then consider the continuation of the project for a further year (if necessary).

This committee abides by the ethical norms and principles for research, established by the Declaration of Helsinki and the Guidelines for Ethical Research: Principles Structures and Processes 2004 (Department of Health). Annually a number of projects may be selected randomly for an external audit.

National Health Research Ethics Committee (NHREC) registration number REC-050411-032.

We wish you the best as you conduct your research.

If you have any questions or need further help, please contact the REC office at .

Included Documents:
ICF Patient Journeys
ICF Waiting Room Patients
Cover Letter: Response to Modifications
Project Proposal
REC feedback letter_MODIFICATIONS.pdf
REC: Humanities New Application

Sincerely,

Clarissa Graham
REC Coordinator
Research Ethics Committee: Human Research (Humanities)

Investigator Responsibilities

Protection of Human Research Participants

Some of the general responsibilities investigators have when conducting research involving human participants are listed below:

1.Conducting the Research. You are responsible for making sure that the research is conducted according to the REC approved research protocol. You are also responsible for the actions of all your co-investigators and research staff involved with this research. You must also ensure that the research is conducted within the standards of your field of research.

2.Participant Enrollment. You may not recruit or enroll participants prior to the REC approval date or after the expiration date of REC approval. All recruitment materials for any form of media must be approved by the REC prior to their use. If you need to recruit more participants than was noted in your REC approval letter, you must submit an amendment requesting an increase in the number of participants.

3.Informed Consent. You are responsible for obtaining and documenting effective informed consent using **only** the REC-approved consent documents, and for ensuring that no human participants are involved in research prior to obtaining their informed consent. Please give all participants copies of the signed informed consent documents. Keep the originals in your secured research files for at least five (5) years.

4.Continuing Review. The REC must review and approve all REC-approved research proposals at intervals appropriate to the degree of risk but not less than once per year. There is **no grace period.** Prior to the date on which the REC approval of the research expires, **it is your responsibility to submit the continuing review report in a timely fashion to ensure a lapse in REC approval does not occur.** If REC approval of your research lapses, you must stop new participant enrollment, and contact the REC office immediately.

5.Amendments and Changes. If you wish to amend or change any aspect of your research (such as research design, interventions or procedures, number of participants, participant population, informed consent document, instruments, surveys or recruiting material), you must submit the amendment to the REC for review using the current Amendment Form. You **may not initiate** any amendments or changes to your research without first obtaining written REC review and approval. The **only exception** is when it is necessary to eliminate apparent immediate hazards to participants and the REC should be immediately informed of this necessity.

6.Adverse or Unanticipated Events. Any serious adverse events, participant complaints, and all unanticipated problems that involve risks to participants or others, as well as any research related injuries, occurring at this institution or at other performance sites must be reported to Malene Fouch within **five (5) days** of discovery of the incident. You must also report any instances of serious or continuing problems, or non-compliance with the RECs requirements for protecting human research participants. The only exception to this policy is that the death of a research participant must be reported in accordance with the Stellenbosch Universtiy Research Ethics Committee Standard Operating Procedures. All reportable events should be submitted to the REC using the Serious Adverse Event Report Form.

7.Research Record Keeping. You must keep the following research related records, at a minimum, in a secure location for a minimum of five years: the REC approved research proposal and all amendments; all informed consent documents; recruiting materials; continuing review reports; adverse or unanticipated events; and all correspondence from the REC

8.Provision of Counselling or emergency support. When a dedicated counsellor or psychologist provides support to a participant without prior REC review and approval, to the extent permitted by law, such activities will not be recognised as research nor the data used in support of research. Such cases should be indicated in the progress report or final report.

9.Final reports. When you have completed (no further participant enrollment, interactions, interventions or data analysis) or stopped work on your research, you must submit a Final Report to the REC.

10.On-Site Evaluations, Inspections, or Audits. If you are notified that your research will be reviewed or audited by the sponsor or any other external agency or any internal group, you must inform the REC immediately of the impending audit/evaluation.
