# Supplementary material for: SMS nudges as a tool to reduce tuberculosis treatment delay and pretreatment loss to follow-up. A randomized controlled trial
Source: PLoS One. 2019 Jun 20;14(6):e0218527. doi: 10.1371/journal.pone.0218527 (PMC6586322; doi:10.1371/journal.pone.0218527)
Supplement: S2 File — (PDF) [file pone.0218527.s002.pdf]

Updated By: Rochelle R Jacobs @ 19-Oct-2016 12:50:50 PM

# Application Form for Ethics Approval



UNIVERSITEIT•STELLENBOSCH•UNIVERSITY
jou kennisvennoot • your knowledge partner

## 1. Application Guidelines

1. All sections must be completed and all information must be included in the form.

2. Please note that all fields marked with a red asterisk (*) are mandatory.

3. The application must be written simply and briefly, providing adequate information for expert review but also at the same time being understandable to lay persons.

4. Please keep the REC deadline dates as published on the website of the Division for Research Development (DRD) in mind for timely submission of your REC application. DESC deadlines are available from your academic department.

5. Please familiarised yourself with the Standard Operating Procedures and Guidelines so that you understand your obligations in terms thereof.

6. Before you begin completing this form please consult the list of documents to be included .

7. In fields that are not mandatory and are not applicable type in "not applicable" and provide motivation for your answer.

9. Sentences in red, highlight things you need to know or consider when completing a question.

10. Sentences in blue, provide instructions on how to use the form.

11. You must consult the InfoEd Manual for Researchers when completing this form.

☑ *I have read the above guidelines

*Enquiries on any REC: Humanities related matters may be directed to the Secretary, Ms Clarissa Graham at +27 21 808-9183 or cgraham@sun.ac.za*

Click here to view the submission closing dates and meeting dates for REC: Humanities

## 2. Does my research qualify for exemption?

Yes ☑ No ☐ *1. My research involves direct interaction with, or data gathering from human participants as individuals, members of a group, organisation or institution (this includes completion of surveys and observation.)

Yes ☑ No ☐ *2. My research involves and requires access to institutional/organisational information or archival data/ archives that are not in the public domain?

Yes ☑ No ☐ *3. My research involves accessing information from a database that contains information linked to personal identifiers (Names, ID numbers, student numbers etc.) OR the database contains coded information but I have access to the code that links the information to identifiers OR the database that I am using is not in the public domain.

Yes ☑ No ☐ *4. My research involves information that is in the public domain but that could be regarded as sensitive, or potentially sensitive?

One or more YES answers? Complete the the rest of this form and submit it.

Only NO answers? The project probably does not require ethics approval (unless it involves animals, environmental or biosafety issues); you need only complete all the compulsory questions and submit the form. Check with your supervisor.

*Please note that should the nature of your data collection method change during your project i.e. one of the answers above becomes YES, you are required to complete the entire e-form and to notify the DESC of this change.*

NB! Please ensure that all required 'permissions' are obtained, if applicable, before starting the study even if ethics approval is not required.

*5. Please upload your research proposal.

# 3. General - proposal details

*1. Title of research project:
Social and Psychological/Behavioral Factors Influencing Non-Initiation of TB Treatment

*2. Please provide a short summary of the proposed research (500 words), that should contain the following:
- A short introduction and motivation
- Research questions or hypotheses
- Study aims and objectives
- Concise summary of the methodology

The research to be conducted explores the behavioral, economic and psychological factors, which may explain non-initiation of tuberculosis (TB) treatment. TB remains a high-priority health concern in South Africa and non-initiation of treatment is of particular concern as it contributes to both the prevalence and incidence of TB.

Research has primarily focused on non-adherence of TB treatment with relatively little account being given to non-initiation of treatment. Where the latter has been considered, it has primarily been from the perspective of supply-side constraints (i.e. waiting times at clinics, quality of healthcare etc.). This study proposes a consideration of the demand-side constraints, which may contribute to non-initiation. More importantly, it considers those hypotheses (namely, those presented by behavioral economics), which are likely to be amenable to low-cost interventions (so-called "nudges").

The study will be conducted by means of retrospective patient journeys that are intended to improve the researchers understanding of the psychological decision-making processes of TB-presumptive patients. Furthermore, surveys will be conducted at 10 high-burden TB clinics in Cape Town and the survey information linked to clinic-level data so as to identify non-initiators.

*3. When do you plan to start your data collection? (date format e.g. 14-Jul-2014) 01-Jan-2017

*4. Data collection end date (date format e.g. 14-Jul-2014) 31-Mar-2017

## 3.1 Data collection commencement

Yes ☐ No ☑ *1. Has data collection already commenced?

## 4. Keywords

Click on the yellow + to add Keywords, then click on the edit icon. To Select a word from the list you must select the word, click on Select and then click on Save.

*Please select 3 keywords or phrases from this list that can be attributed to your study. Please note you can only select 1 word from the list at a time. Before you can add an additional keyword you must click on the yellow plus this must be done before every addition.

- Health Care Economics
- Behavioral/Experimental Psychology
- Behavior and Health

## 4.1 Keywords not listed above

Yes ☑ No ☐ *1. Were you able to find all the keywords in the above list?

## 5. Investigator information

*1.Is this research for degree purposes?
Yes ☑ No ☐

2. What degree?
Masters in Economics

*3. Name of institution where degree will be obtained
Stellenbosch University

NB! PLEASE NOTE IF YOU ARE NOT CURRENTLY A SCHOOL OF PUBLIC LEADERSHIP (SPL) REGISTERED STUDENT OR STAFF MEMBER TO QUESTION 5 PLESASE SELECT “NO” AND QUESTION 6 MUST REMAIN BLANK.*IF THIS IS COMPLETED INCORRECTLY YOUR FORM WILL BE SENT TO THE WRONG DESC WHICH WILL RESULT IN A DELAY IN THE REVIEW OF YOUR APPLICATION.*

*5. Are you based at the School of Public Leadership (e.g. a registered student or staff member at SPL)? No

*If this study is for degree purposes, please ensure that you add your supervisor's details under Section 5.1. Stellenbosch University Investigators*

Please Note

1)Students not yet registered or Stellenbosch University affiliated persons (eg. staff or external supervisors) not listed on the Human Resource system or SunID database will not be able to login to InfoEd and will not be listed in the personnel drop down list below in section 5.1.

2)If you cannot find a student, staff member or affiliated person in Section 5.1 please make sure with your department that the person(s) has been added to one of the official university systems (Student Information Systems (i.e. a registered student) or HR, or SunID). If they aren't your department will first have to add them before you can add them to your application.

3)Students who wish to apply for ethics clearance before they are registered must do this in consultation with their relevant department. The department will have to add them to the Information Technology SUNID temporary user database so that the student can be issued a username and password by IT. Please note that the SUNID process in not an ethics process, it is a completely separate process and is handled entirely by the relevant department/ division and the IT helpdesk.

4) CHECK STELLENBOSCH UNIVERSITY STAFF AND STUDENT CONTACT DETAILS:Please note that you must check the information in the column “Name and Surname” for every person added. Should you encounter any inaccuracies in the contact information (e.g. email, department etc.) displayed in the table below please contact your Human Resources Practitioner for staff members and Student Information for students to have it corrected before continuing with this form. DO NOT SUBMIT THIS APPLICATION BEFORE THE INFORMATION HAS BE BEEN CORRECTED. We rely solely on this information when sending correspondence and information may not reach you if the contact details are not correct.

5) If you are a student please make sure you add your supervisor. Please note that the list of information in 5.1 comes from our official sources and therefore your Supervisor will be listed by their surname and their first name which may differ to the name they use in their day to day interactions so please make sure you have their full name before you start the application. If your supervisor is not listed please contact your department/ division to ensure that your supervisor (especially in the case of an external supervisor ) has been added either on HR or SunID by your department/ division before you submit this application.

6) Note: If you selected a person by mistake, you can make use of the remove button (blue arrow) next to the person's name and then add the correct name. If you added a row and want to delete the entire row, click the Delete button (bin icon) .

7) Should you not find Stellenbosch University Investigators or students please also notify Lee Louw (leelouw@sun.ac.za/021 808 9444/ 021 938 9092) or Nicole Walker (nwalker@sun.ac.za/ 021 808 4914).

### 5.1. Stellenbosch University Investigators (Please click on the yellow "+" in the right hand corner to add people) (Fields that are not applicable can be left blank)

| *Internal Personnel | *Highest qualification | *Professional registration | *Registration number | *Role in project | Cell number |
|---|---|---|---|---|---|
| Burger, Ronelle R<br>Full Name: Burger, Ronelle R<br>Address 1:<br>Address 2:<br>City:<br>Zip:<br>Email: rburger@sun.ac.za<br>Phone: 218 083 106<br>Employee/Student ID: SU_12200166<br>Department: Economics | Doctrate | None | | Primary Investigator (PI) | |
| Jacobs, Rochelle R<br>Full Name: Jacobs, Rochelle R<br>Address 1:<br>Address 2:<br>City:<br>Zip:<br>Email: 17066301@sun.ac.za<br>Phone: 218084513<br>Employee/Student ID: SU_17066301<br>Department: Economics | Honours Economics | None | | Post Grad Student PI | 0833917165 |

## 5.2 External Investigators cont.

Yes ☑ No ☐ *1. Are there any personnel external to Stellenbosch University involved in conducting this research?

### 5.2.1 External Personnel (Please click on the yellow "+" in the right hand corner to add people) (Fields that are not applicable can be left blank)

| *Full name | *Highest qualification | Professional registration | Telephone number | *Role in project | *Organisation/ Department | *Position |
|---|---|---|---|---|---|---|
| Pren Naaidoo | Dr | | | Clinical Associate | Desmond Tutu TB Center | TB Consultant |
| Mareli Claasens | Dr | | | Clinical Associate | Desmond Tutu TB Center | Research Clinician |
| Adam Wagstaff | Dr | | | External PI | World Bank | Research Manager |
| Eddy v Doorslae | Dr | | | External PI | Erasmus University Rotterdam | Professor of Health Economics |
| Martin Abel | Masters | | | Sub/Co Investigator | Harvard University | PhD Candidate |
| Carmen Lopes | Master | | | Sub/Co Investigator | University of the Western Cape | Lecturer |
| Judi Cladwell | Dr | | | Clinical Associate | Cape Town Health Directorate | TB Manager |
| Ronelle Burger | Dr | | | Supervisor | University of Stellenbosch | Associate Professor |

## 6. Familiarity with codes of ethical conduct

- [x] *1. I have familiarised myself with the SU Ethics Policies.
- [ ] 2. I have familiarised myself with the professional code(s) of ethics and/or guidelines for ethically responsible research relevant to my field of study.

## 7. Nature of the proposed research

Is the study:

Yes [ ] No [x] *1. Part of a bio-medical research project

Yes [x] No [ ] *2. A multi-institutional project

Yes [x] No [ ] *3. Do any of these institutions require proof of ethics clearance from a registered REC?

Yes [x] No [ ] *4. Funded from sources other than SU or self

Yes [ ] No [x] *5. Does the funder have specific requirements for ethics review e.g. proof of ethics clearance from a registered REC?

REC referral may be required

*6. Please list the funder(s)
World Bank
Gates Foundation

# 8. Participants

Does the study intentionally involve the collection of data on people in the following categories?

☐ Yes ☑ No *1. Minors

☐ Yes ☑ No *2. People living with, or affected by HIV/AIDS

☐ Yes ☑ No *3. Prisoners

☐ Yes ☑ No *4. People living with disabilities

*5.Other category deemed vulnerable (see Glossary in REC SOP)
☑ Yes ☐ No

*5.1. If yes specify:
Persons with tuberculosis

☐ Yes ☑ No *6. Stellenbosch University staff, students, alumni or other persons/organisations associated with the university

## 8.2 Participants continued

Does the proposed research involve processes regarding the selection of participants in the following categories:

Yes ☐ No ☑ *1. Participants that are subordinate to the person doing the recruitment for the proposed research

Yes ☐ No ☑ *2. Third parties that are indirectly involved because of the persons being studied (e.g. family members of HIV patients; parents or guardians of minors, friends)

## 8.3 Participant recruitment

*1. Briefly describe the inclusion and exclusion criteria to be used and explain why they are appropriate to this study (if your sample includes vulnerable sub populations please justify their inclusion and describe safeguards to minimise risk)
All patients in the waiting rooms of TB clinics will be surveyed. This allows us to study the behaviour of both those who are TB positive and those who are not, prior to their knowledge of the fact. This allows us to increase our sample size as well as observe any notable differences.

*2. How will participants be invited to take part in this study, please also specify the time and place where applicable.
Participants will be invited to participate during their wait in the clinic waiting rooms. They will be offered refreshments, but no other compensation, for their efforts. Surveys will be conducted via electronic devices (such as iPads) but field workers will be available to assist, if necessary.

Yes ☐ No ☑ *3. Will an advertisement or flyer be used in recruiting participants for this study?

## 9. Steps to ensure established ethical standards are applied

Yes ☑ No ☐ *1.1. Has provision been made for written informed consent ?

Yes ☐ No ☑ *1.2. Has provision been made for verbal informed consent ?

Please complete the section "Consent forms" below.

Please click here for a template consent form.

Yes ☑ No ☐ *2. Will participant(s) be informed that they have the right to refuse to answer questions?

Yes ☑ No ☐ *3. Will participant(s) be informed that they have the right to withdraw from participation at any time?

Yes ☑ No ☐ *4. Will steps be taken to ensure personal data of informants will be secured from improper access?

*4.1. Explain these steps

All patient level information will be securely stored and only available to the researchers. Since there are no unique patient identifiers, we will use patient names to match data from questionnaires to laboratory test data. On completion of data collection, study data will be anonymised through tokenisation of all personal identifiers in the research data. The token database used for conversion will be securely stored and available only to the study researchers.

Yes ☑ No ☐ *5. Will confidentiality of data be maintained?

*5.1. Explain the process(es) for maintaining confidentiality

Tokenisation of identifying data will be used to convert any personal identifiers. This allows researchers to match and track individuals without their identity being known.

Yes ☑ No ☐ *6. If unexpected, unsolicited data is revealed during the process of research, will data be kept confidential and only revealed if required by law?

Yes ☑ No ☐ *7. Will anonymity of participant(s) be maintained during reporting of results?

*7.1. Describe process(es) for maintaining anonymity

Tokenisation of identifying data will be used to convert any personal identifiers. This allows researchers to match and track individuals without their identity being known.

Yes ☑ No ☐ *8. 1. Will research assistants or fieldworkers be used to collect data?

Yes ☑ No ☐ *8.2. Will ethics awareness be included in their training?

### 9.1 Consent form(s) (Please click on the yellow "+" in the right hand corner to add)

| Participant group | Home language | Specify other home language (if applicable) | *Upload consent form(s) |
|---|---|---|---|
| TB positive patients | | | |
| Waiting room patient | | | |

## 10. Risk of harm

*1. What is the likelihood that mitigation of risk of harm to participants (making use of suitable persons or organisations that are able to offer counselling or assistance to participant(s) during or after the research) will be required? Low

Yes ☑ No ☐ *2. If an unexpected emergency situation is revealed during the research, whether it is caused by your research or not, will it immediately be reported to your supervisor/promoter and/or Departmental Chair for further advice?

## 11. Institutional / Organisational permission

Yes ☑ No ☐ *1. Is institutional/ organisational permission required to gain access to subjects/participants?

Yes ☐ No ☑ 2. Is/Are (a) all permission letter(s) available?

Yes ☐ No ☑ 3. Are only some permission letters available?

Please attach copies of the application letters for institutional permission at section 15.

Yes ☑ No ☐ 5. Do you intend to apply for institutional permission

*Specify from whom:City of Cape Town Health Department

## 12. Data collection instrument

Yes ☐ No ☑ *1. Will (an) existing instrument(s) to which copyright applies, be used to gather data?

Yes ☐ No ☑ *2. Is/are the instruments that will be used to gather data, classified by law as psychological tests?

### 12.2 Method of Data Collection

| *Select all applicable methods | *Specify Other | *Attach Here (if applicable) |
|---|---|---|
| Interview | | |
| Questionnaire | | |

## 13. Conflict of interest

Yes ☐ No ☑ *1. Are you aware of any actual or potential conflict of interest in proceeding with the proposed research?

## 14. Risk classification

*1. Please assess the risk of potential harm as a result of the proposed research (download the DESC guidelines) here. 3. Medium risk

REC referral required

## 15. Additional documents to be uploaded (Please click on the yellow "+" in the right hand corner to add)

| Document Name | Upload |
|---|---|
| Cover Letter | |
| Cover Letter: Response to REC | |

## Translations

If any documents will be translated into any language other than Afrikaans or English attach convincing evidence that the translation is an accurate and complete representation of the original document i.e. statement by a certified language practitioner or equivalent.

## 16. Additional Information

Please provide any additional information that you deem relevant to your application
No translation will be undertaken, based on our
experience with English in clinics thus far.

## 17. Willingness to be contacted for research purposes

The Research Ethics Committees: Humanities at Stellenbosch University is part of the academic complex. As such vital research around research and ethics of research is encouraged. Please indicate your willingness to participate in such research by answering the following questions:

Yes ☑ No ☐ *1. May the information provided in this application be used in research around research and ethics?

Yes ☑ No ☐ *2. May approved researchers contact you regarding the information submitted here?

## Submiting this application

1. Please remember that in order to complete this form you must complete all the mandatory sections, scroll to the top of this page and then tick the complete box. To save a copy of this application click on the PDF icon on the top left of the form. Then click on the red cross and the form will then be closed.

2. Only tick Complete once you are done with the form. Please note that every time you tick complete (i.e. click on complete then untick complete (to make edits) and then click on complete again) a new version of the form is created. So please only click on complete when you are sure you are done with the form in order to control the number of versions created.

3.Please note that completing this form does not mean it has been submitted you must still submit this application on the InfoEd Components of Initial Application Screen to submit this submission to the HREC, this is the page where you added the eform on. If this page is closed please follow the steps in the manual on how to retrieve your form.

For further instructions on this please consult the InfoEd Manual for Researchers or contact Clarissa Graham cgraham@sun.ac.za

# Appendix 1

**EForm Name**: REC: Humanities New Application
**Page**: Page 2
**Section**: 2. Does my research qualify for exemption?
**Question**: 5. Please upload your research proposal.
**File Name**: LPA_Non_Initiation_161018.docx

# Social and Psychological/Behavioral Factors Influencing Non-Initiation of TB Treatment

~~Understanding non-initiation of TB treatment amongst TB testers~~

### Preliminary analysis on TB treatment non-initiation rates

Prevoius studies show rates of 20-25% for five sub-districts in Cape Town. However, this analysis has limitations as it is based on retrospective, routine data; the completeness of routine data is difficult to assess from currently available information. Some non-initiation may be attributable to reporting error rather than non-initiation. Whilst we are able to quantify non-initiation, we also have no information on why it occurs and whether there are cost-effective interventions that can help address the problem.

The current results suggest a significant problem, with serious consequence for patient morbidity and mortality and ongoing TB transmission in communities. It is important to understand the issue of non-initiation in more depth and specifically to explore the motivation behind individuals' choices.

We propose to provide more in-depth information that can be used to develop an intervention to address the problem in the future. The study will be undertaken by Rochelle Jacobs, Masters student in Economics at Stellenbosch University, and will be supervised by Prof Ronelle Burger, Department of Economics, Stellenbosch University.

### Outline of study: Who returns to the clinic to collect test results after a TB test?

### 1. Background

A non-initiator[1] is a person with presumptive TB who is recorded as bacteriologically-positive but who is not recorded in the TB register where TB treatment is recorded and monitored (Harries, Rusen et al. 2009); the presumption is that such an individual has not initiated TB treatment. This definition is fairly broad and non-specific, and does not stipulate a cut-off time for the period between diagnosis at the facility and commencement of treatment (recorded in TB register). The cut-off period is left to the discretion of the researcher and may therefore differ between various non-initiation studies (cf. Botha et al. 2008b; Claassens et al. 2016). In this proposal we define non-initiators as patients who do not appear in the TB treatment register[2] within four weeks of bacteriological confirmation – via GeneXpert, smear or culture – at the primary health care facility. Many non-initiators never return to the facility to get their test result.

While non-initiators are defined only within the sample of TB disease positives, we argue here that the most important question to examine may be why many individuals who test themselves for TB never return to the clinic to learn the results of their TB tests.

### The scale of non-initiation of TB treatment in South Africa and elsewhere

A 2009 evaluation of South Africa's TB Surveillance System reported that 33.7% of all persons suspected with TB who were recorded as smear positive in the TB Suspect Register were not picked up in the TB treatment register (Podewils et al. 2015). Hence up to one third of the sample reviewed were considered non-initiators, a missed opportunity for TB control considering that persons with presumptive TB already accessed the public health system but were not treated. This finding not only highlighted the

---

[1] Our preferred term to describe 'initial defaulters' or 'initial loss to follow-up patients'.
[2] It is assumed this patient has not initiated treatment.

importance of monitoring the integrity of surveillance information but also provided an estimate of the magnitude of the non-initiator problem in South Africa. A comparable study in Botswana showed that 27% of confirmed smear-positive TB patients had either no evidence of treatment initiation or treatment delay (Creek et al. 2000).

Smaller scale research in Western Cape Province of South Africa cite a non-initiator rate of 16% (58/373) in a Cape Town study (Botha et al. 2008b) and 17% (64/367) in a Stellenbosch study (Botha et al. 2008a). These figures are comparable with those reported in rural Malawi (15%) (Squire et al. 2005). Den Boon et al. (2008) explored active case detection in two neighboring suburbs in Western Cape Province and found a non-initiator rate of 26% (7/27). In a 2009 study conducted at 122 primary health care facilities in five provinces, Claassens et al. (2016) find a mean non-initiation rate of 25% (95% CI 22-28) across provinces. These figures compare to non-initiator rates for active case findings in South India (32%) (Santha et al. 2003) and a regional hospital in Ghana (38%) (Afutu et al. 2012).

It should be noted that non-initiators are not recorded in the TB Treatment Register, and therefore do not feature in TB outcome statistics. South Africa's official statistics thus overestimate the country's treatment success rate (Botha et al. 2008a; Harries et al. 2009). Correct interpretation of national data on TB incidence and trends requires that non-initiator rates, amongst other factors, are accurately captured and adequately understood (Migliori et al. 1995).

### Causes of non-initiation of treatment in South Africa and elsewhere

Reasons for non-initiation of treatment are unclear, and there is a relatively small literature on the subject, even internationally; furthermore, the existing literature is invariably based on small samples. A systematic review and meta-analysis of non-initiators in low- and middle-income, high-burden countries (MacPherson et al. 2014) could identify only 23 eligible studies, eight of which were conducted in Africa, of which four were from South Africa.

Most studies recognize that the reasons for non-initiation could stem from demand-side factors (i.e. linked to the patient), supply-side factors (i.e. linked to the health care system), or a combination of the two. Studies rarely give a sense of which matter more.

An exception is a 2005 study by Botha et al. (2008b) exploring the nature of non-initiators in Cape Town. The authors found that among those who could be traced and interviewed (24% had died, and 45% could not be traced), 56% cited the main reasons for non-initiation being directly linked to the health care system, while 44% did not. By contrast, a related study conducted by Botha et al. (2008a) in the Stellenbosch area was unable to clearly identify reasons for non-initiation.

Despite lack of clarity as to their importance in explaining non-initiation, supply-side factors linked to NTP system errors and health systems gaps have dominated the literature to date. These include weaknesses in the systems between the laboratory, where TB is bacteriologically diagnosed, and the primary health care facility, where the patient was tested (Edginton et al. 2005; Botha et al. 2008a; Botha et al. 2008b; Claassens et al. 2013b), as well as non-reconciliation and lack of monitoring of various TB registers (Sai Babu et al. 2008; Harries et al. 2009). Suggestions of simple health system interventions – such as noting the correct address and contact details to facilitate follow-up – are proposed to reduce the number of non-initiators (Harries et al. 2009; Dunbar et al. 2011; Afutu et al. 2012).

Understanding the health-seeking behavior of non-initiators and the specific characteristics associated with it has been the subject of very little research. It is known that a considerable fraction of people infected with TB – many of whom are co-infected with HIV – seek care for TB at a very advanced stage of their disease (Botha et al. 2008b; Sai Babu et al. 2008). Some of the non-initiators die shortly after being tested – either at home or in a hospital – and this information is never recorded in the TB Register (Edginton et al. 2005; Podewils et al. 2015).

Reasons why patients never return to get their test results are not well understood. Studies suggest some factors. Some cite lack of health education and poverty as correlates of non-initiation (Edginton et al. 2006; Munir et al. 2012). With patients incurring the greatest share of TB episode costs (41%) prior to commencing treatment, indirect costs (the largest portion being loss of income) are also thought to be a potential barrier to TB treatment for the poor (Foster et al. 2015).

Fear of stigmatization, including being considered HIV-positive, may also feed into non-initiation of treatment (Cramm et al. 2010; Murray et al. 2012); this is supported by recent in-depth interviews with 41 non-initiator and initiator respondents across 5 provinces in South Africa by Skinner & Claassens (2016). Murray et al. (2012) also cite the association of dirt and squalor with TB as impeding TB diagnosis, and comment on how this fuels a sense of disempowerment felt by members of poorer communities, where contracting TB is perceived as inevitable; this disempowerment, in turn, feeds into an apathy for seeking treatment.

Among TB patients identified via active case detection, it is speculated that a lack of motivation to start treatment may be attributable to the absence of, or less severe, TB symptoms (Den Boon et al. 2008); there is some evidence to support this in the recent work of Claassens et al. (2016) who found non-initiators were more likely to have 'scanty'[3] smears. However, Claassens et al. (2016) also note that there is a potential supply-side angle to this explanation: health workers may not be aware that scanty smear-positive individuals should start treatment, and that this sub-group is neglected when tracing TB patients in the community. Skinner & Claassens (2016) also find patient knowledge and beliefs to be a factor in non-initiation, along with the perceived unaffordability of health care, and complaints about the health system – lack of resources, poor organization, and negative staff attitudes.

## 2. Study methods

### Aims and Objectives

To complement existing analysis on loss to follow up using the routine data, we will conduct a survey to gather additional data on individual characteristics, perceptions, beliefs and attitudes of those testing for TB.

Our aim is to understand how these individual level variables are correlated to the choice of returning to the clinic to collect TB test results.

After conducting the survey, we will match individuals to registers to ascertain who tested positive and who returned to the clinic to collect their results within the window period.

[3] A 'scanty' smear is a sputum smear positive which has less than 10 acid fast bacilli per 100 high power fields,

## Research questions and hypotheses

The preliminary review of the literature above suggests two broad sets of economics-related hypotheses that might explain non-initiation of TB treatment.

Individuals often fail to initiate treatment at the very first step—they never return to the facility to get their TB test result. These people may think (rightly or wrongly) they are not likely to have TB and therefore the benefits are likely to be small. Or they may think (rightly or wrongly) that the likely benefits from treatment are relatively low because they (incorrectly) believe that TB is not curable (Murray et al. 2013). Or non-initiators may believe they are sick and know TB is curable but have less confidence in the health system to deliver the necessary care, because of drug stock-outs, lack of competence of health workers, etc.

Non-initiators may also be concerned (rightly or wrongly) about the time costs of the course of treatment. They may not be fully informed about the switch from the directly-observed treatment approach, which entails large time costs for the patient including actual forgone earnings (Ukwaja et al. 2012). Or they may be aware of the switch, but realize there are still some time inputs required with the community-based approach (Foster et al. 2015): non-initiators may attach a higher value to their time.

Lastly, non-initiators may be concerned (rightly or wrongly) about the monetary costs of the course of treatment. They may not realize that TB treatment in South Africa is free in public facilities (Foster et al. 2015) and may expect the impact on household consumption to be larger than it is: non-initiators may be less well informed about the out-of-pocket costs of TB treatment. On the other hand, non-initiators may realize that there are indeed significant direct costs such as the costs of foodstuffs which are apparently especially large during the pre-diagnosis and diagnostic phase (Foster et al. 2015): non-initiators may be poorer and less able to afford these costs. Or non-initiators may be put off by the transport expenditures (Foster et al. 2015): non-initiators may face worse access to health facilities.

However, it is important to also consider that people's behavior in the face of illness may not always conform to the rationality ascribed by economists to *Homo Economicus*. People take decisions factoring in what others around them think and do—the World Development Report calls this "thinking socially". One example of such an influence is stigma, which is thought to affect people's choices vis-à-vis TB treatment (Murray et al. 2013): TB stigma has intensified due to its linkages to HIV/AIDS,  and non-initiation of treatment may be due to fear of being stigmatized, either in the community or at work. In South Africa, this fear is likely compounded by the low confidence that South Africans appear to have in patient confidentiality (Murray et al. 2013). Non-initiators may have less confidence in the system's protection of patient confidentiality and/or have a greater concern about stigma.

The World Development Report also highlights the tendency for the quality of decision-making to fall when people's quality cognitive resources are 'taxed'. As the World Development Report puts it, "When cognitive resources are overtaxed, decision quality typically suffers, as decisions are driven by emotional impulses and a narrow short-term focus." (2015) When illness is just one of many challenges a family faces, along with poverty, the threat of crime, the fear of job loss, and other stressors, going to the facility to collect the TB test result and initiate treatment may well not be the action at the top of the family's to-do list. Non-initiators may have a longer list of competing stressors in their lives.

Behavioral economics also predicts that the outcome of any given decision is altered by the context in which it is made and the information or reference point to which people compare their own circumstances. This process of anchoring may manifest itself in many ways and a number of these may explain non-initiation of treatment. This applies to non-initiation in terms of perceptions on treatment versus illness (do people 'anchor' their decision in terms of the cost of treatment and not the cost of remaining ill?), concepts of social responsibility (do treatment initiators consider the benefit to their communities more than non-initiators do?) and 'present bias' (do non-initiators discount their time-preferences more highly?). These hypotheses are based on fundamentally psychological insights and combine with economics in terms of their mapping to cost-benefit analysis.

### Participant recruitment

Presumptive TB cases i.e. individuals presenting with TB symptoms and having sputum tests submitted for TB testing at the laboratory will be eligible. Only individuals over 18 years in age will be eligible. The survey will be conducted in ten high-burden facilities in Cape Town (Manenberg, Eastridge, Nyanga, Lentegeur, Weltevreden Valley, Chapel Streel, Philani, Bloekombos, Ikwezi, Delft South Clinics). ~~where we have permission from the City Health Directorate to undertake in-depth work.~~

Nurses at each facility will ask eligible individuals if they are willing to participate and refer these individuals to the field researcher. The field researcher will go through the informed consent process with each individual (see Annexure A1 and A2) and guide participants through the completion of the questionnaire (Annexure B).

There are currently 1700 presumptive TB cases tested per month in these facilities. Allowing for non-participation, we expect to sample about 3 000 individuals over two months. Data on the age and gender of non-participants will be collected to assess representativeness of participants.

### Questionnaire administration

We will survey presumptive TB cases via a self-administered questionnaire while they are at the facility. This sampling strategy is motivated by four considerations:

1) This strategy avoids any losses due to records not captured in the registers or not captured fully (address, cellphone number) in the administrative records

2) This strategy is expected to minimizes refusals given that the cost of an individual's time may be comparatively low if we can talk to them while they are at the clinic

3) We need to obtain their individual consent to access their records on the register

4) This avoids ex-post rationalization and retrospective self-justification, which is expected to play a large role if returning to the clinic is not a conscious rational choice, or may have irrational reasons or components that individuals are ashamed to reveal.

We plan to put the survey on an electronic device so that respondents can complete it themselves, in the language of their choice. If respondents are not literate, have low literacy or have difficulty in understanding questions, a field researcher will be available to assist participants.

The questionnaire will be piloted thoroughly to ensure that all questions are easily understandable and to ensure that it is not too long and imposes a reasonable time burden on respondents. Surveys that are too long may result in respondent fatigue setting in towards the end of the questionnaire, which can lead to a lower quality of answers and lower reliability for the last segments of the questionnaire.

Participants will not be financially compensated for their participation, but will be provided with refreshments.

### Retrospective Patient Journeys

Preceding the survey questionnaire in the clinic waiting rooms, we will also conduct 60 retrospective patient journeys – 30 in the Western Cape and 30 in the Eastern Cape. These will take the form of interviews and questions about how patients were diagnosed, the manner in which they began treatment and the treatment process itself. Patients will be identified through the use of patient records from the ETR.net database and select patients that are at the end of their treatment journeys. Selected patients will be stratified by treatment outcome categorization.

### Assessing the potential effect size of information interventions

Interventions may take three forms: information interventions during questionnaire administration, cost interventions by setting appointments for collecting test results and sms reminders. In the case of information interventions, behavioral economics indicates that there are a number of ways in which information may be inaccurately recalled or processed by individuals in high-stress decision contexts. Information interventions, aimed at correcting such biases, may be included at the end of the questionnaires.

Similarly, present-biased preferences are often overcome by adjusting the cost or benefit of a later decision (such as returning to the clinic to collect test results). A low-cost intervention to reduce the perceived cost of returning to collect test results may take the form of an appointment card, if the patient is willing to specify the day on which he/she will return.

After 28 days has passed the team will send sms reminders to a random sample of those who have consented to follow-up contact and who have not returned to the clinic. This aims to provide an estimate of the potential effect of a low cost and lightweight intervention with wide reach. The proposed content of the sms message will be finalised with the DOH.

### Data collection and management

Questionnaire data will be completed directly in password protected electronic devices and downloaded on a weekly basis onto a secure back-up. Each completed questionnaire will be saved on a password protected data file, accessible only to study researchers.

We will collect information about test results and whether participants returned to the clinic by matching study information with patient data from the electronic PREHMIS system that is used to collect routine visit data in facilities. We will use laboratory faxed results sheets to confirm TB test results.

Laboratory data on test results (from fax sheets) will be collected into an access database at each facility.

Since the Health Department does not use unique patient identifiers, matching study participants to these data sources may be challenging. Field researchers will take all necessary precautions by gathering adequate demographic information about each individual respondent and by matching data on weekly basis at each facility.

All patient level information will be securely stored and only available to the researchers. Since there are no unique patient identifiers, we will use patient names to match data from questionnaires to laboratory test data. On completion of data collection, study data will be anonymised through tokenisation of all personal identifiers in the research data. The token database used for conversion will be securely stored and available only to the study researchers.

### Data analysis

Participant demographic characteristics will be analyzed using the t-test for normally distributed continuous outcomes and chi-square for categorical outcomes. Multivariable regression analysis will be used to identify factors associated with non-return to health facilities and with TB treatment non-initiation. Analyses will be undertaken using STATA 12 (StataCorp).

## 3. Study management

Prof Ronelle Burger (Department of Economics) will assume overall responsibility for this study.

Dr Pren Naidoo, the PI of the parent study will provide guidance where required.

Rochelle Jacobs, a Masters student from the Department of Economics will be responsible for the day to day management of field researchers and for data management.

A number of health economists with substantial knowledge of TB in the South African context will also be assisting on the project. These include Prof. Adam Wagstaff, of the World Bank, Prof. Eddy van Doorslaer from Rotterdam University and Carmen Sue Sue Christian Lopez, a PhD student under supervision by Prof. Ronelle Burger.

Further assistance will be given by Dr. Judy Caldwell, Dr. Mareli Claassens and Martin Abel.

Field researchers will be responsible for getting informed consent from participants, assisting with completion of questionnaires, collection of information on return visits and test results, and entering test and participant visit data into the access database.

## 4. Budget

Field work costs are estimated to be about R400 per day (including an allowance for phone calls and travel) for 12 field workers for 40 days, amounting to R192 000.

A small bursary of R40 000 will be paid to a Masters student working on the project.

R30,000 (R10 per participant) has been budgeted for refreshments.

Researchers on the team are all donating their research time to the project at no cost.

The questionnaires will be completed on available tablet/Ipad/handheld devices and thus we do not include a budget for data entry or for hardware.

## 5. References and relevant literature informing this design


Afutu, F. K., R. Zachariah, S. G. Hinderaker, H. Ntoah-Boadi, E. A. Obeng, F. A. Bonsu and A. D. Harries (2012). "High initial default in patients with smear-positive pulmonary tuberculosis at a regional hospital in Accra, Ghana." Transactions of the Royal Society of Tropical Medicine and Hygiene **106**(8): 511-513.

Atkins, S.-A. A. (2011). Improving adherence: An evaluation of the enhanced tuberculosis adherence model in Cape Town, South Africa, Inst för folkhälsovetenskap/Dept of Public Health Sciences.

Atkins, S., S. Lewin, K. C. Ringsberg and A. Thorson (2012). "Towards an empowerment approach in tuberculosis treatment in Cape Town, South Africa: a qualitative analysis of programmatic change." Global health action **5**.

Baeten, S., T. Van Ourti and E. van Doorslaer (2013). "Rising Inequalities in Income and Health in China: Who Is Left Behind?" Journal of Health Economics **32 6**: 1214-1229.

Becker, G. (1964). Human Capital: A Theoretical and Empirical Analysis With Special Reference to Education. New York, National Bureau of Economic Research.

Becker, G. (1965). "A theory of the allocation of time." Economic Journal **75**: 492-517.

Bonfrer, I., R. Soeters, E. Van de Poel, O. Basenya, G. Longin, F. van de Looij and E. van Doorslaer (2014a). "Introduction of performance-based financing in burundi was associated with improvements in care and quality." Health Aff (Millwood) **33**(12): 2179-2187.

Bonfrer, I., E. van de Poel, M. Grimm and E. Van Doorslaer (2014b). "Does the Distribution of Healthcare Utilization Match Needs in Africa?" Health Policy and Planning **29 7**: 921-937.

Bonfrer, I., E. Van de Poel and E. Van Doorslaer (2014c). "The effects of performance incentives on the utilization and quality of maternal and child care in Burundi." Soc Sci Med **123**: 96-104.

Botha, E., S. Den Boon, K. A. Lawrence, H. Reuter, S. Verver, C. J. Lombard, C. Dye, D. A. Enarson and N. Beyers (2008a). "From suspect to patient: tuberculosis diagnosis and treatment initiation in health facilities in South Africa." The International Journal of Tuberculosis and Lung Disease **12**(8): 936-941.

Botha, E., S. Den Boon, S. Verver, R. Dunbar, K. A. Lawrence, M. Bosman, D. A. Enarson, I. Toms and N. Beyers (2008b). "Initial default from tuberculosis treatment: how often does it happen and what are the reasons?" The International Journal of Tuberculosis and Lung Disease **12**(7): 820-823.

Burger, R. (2005). "What We Have Learnt from Post-1994 Innovations in Pro-poor Service Delivery in South Africa: A Case Study-Based Analysis." Development Southern Africa **22 4**: 483-500.

Burger, R. and T. Owens (2010). "Promoting Transparency in the NGO Sector: Examining the Availability and Reliability of Self-Reported Data." World Development **38 9**: 1263-1277.

Burger, R. (2011). "School Effectiveness in Zambia: The Origins of Differences between Rural and Urban Outcomes." Development Southern Africa **28 2**: 157-176.

Burger, R., C. Bredenkamp, C. Grobler and S. van der Berg (2012a). "Have Public Health Spending and Access in South Africa Become More Equitable since the End of Apartheid?" Development Southern Africa **29 5**: 681-703.

Burger, R. and T. Owens (2013). "Receive Grants or Perish? The Survival Prospects of Ugandan Non-governmental Organisations." Journal of Development Studies **49 9**: 1284-1298.

Burger, R., I. Dasgupta and T. Owens (2015a). "A Model of Nongovernmental Organization Regulation with an Application to Uganda." Economic Development and Cultural Change **64 1**: 71-111.

Burger, R., I. Dasgupta and T. Owens (2015b). "Why Pay NGOs to Involve the Community?" Annals of Public and Cooperative Economics **86 1**: 7-31.

Burger, R. P., R. Burger and L. Rossouw (2012b). "The Fertility Transition in South Africa: A Retrospective Panel Data Analysis." Development Southern Africa **29 5**: 738-755.

Capuno, J. J., A. D. Kraft, S. Quimbo, C. R. Tan, Jr. and A. Wagstaff (2015). "Effects of Price, Information, and Transactions Cost Interventions to Raise Voluntary Enrollment in a Social Health Insurance Scheme: A Randomized Experiment in the Philippines." Health Econ.

Christian, C. S. and N. Crisp (2012). "Management in the South African public health sector: An x-inefficiency perspective." Development Southern Africa **29**(5): 725-737.

Claassens, M., C. Van Schalkwyk, L. den Haan, S. Floyd, R. Dunbar, P. Van Helden, P. Godfrey-Faussett, H. Ayles, M. Borgdorff, D. Enarson and others (2013a). "High prevalence of tuberculosis and insufficient case detection in two communities in the Western Cape, South Africa." PloS one **8**(4): e58689.

Claassens, M. M., E. Du Toit, R. Dunbar, C. Lombard, D. A. Enarson, N. Beyers and M. W. Borgdorff (2013b). "Tuberculosis patients in primary care do not start treatment. What role do health system delays play?" The International Journal of Tuberculosis and Lung Disease **17**(5): 603-607.

Claassens, M. M., E. Jacobs, E. Cyster, K. Jennings, A. James, R. Dunbar, D. A. Enarson, M. W. Borgdorff and N. Beyers (2013c). "Tuberculosis cases missed in primary health care facilities: should we redefine case finding?" The International Journal of Tuberculosis and Lung Disease **17**(5): 608-614.

Claassens, M. M., R. Dunbar, B. Yang and C. J. Lombard (2016). Scanty smears associated with initial loss to follow-up in South African TB patients.

Colvin, C. J., N. Leon, C. Wills, M. van Niekerk, K. Bissell and P. Naidoo (2015). "Global-to-local policy transfer in the introduction of new molecular tuberculosis diagnostics in South Africa." The International Journal of Tuberculosis and Lung Disease **19**(11): 1326-1338.

Cramm, J. M., H. J. M. Finkenflügel, V. Møller and A. P. Nieboer (2010). "TB treatment initiation and adherence in a South African community influenced more by perceptions than by knowledge of tuberculosis." BMC public health **10**(1): 1.

Creek, T. L., S. Lockman, T. A. Kenyon, M. Makhoa, N. Chimidza, T. Moeti, B. B. Sarpong, N. J. Binkin and J. W. Tappero (2000). "Completeness and timeliness of treatment initiation after laboratory diagnosis of tuberculosis in Gaborone, Botswana." The International Journal of Tuberculosis and Lung Disease **4**(10): 956-961.

Den Boon, S., S. Verver, C. J. Lombard, E. D. Bateman, E. M. Irusen, D. A. Enarson, M. W. Borgdorff and N. Beyers (2008). "Comparison of symptoms and treatment outcomes between actively and passively detected tuberculosis cases: the additional value of active case finding." Epidemiology and infection **136**(10): 1342-1349.

du Toit, E., S. B. Squire, R. Dunbar, R. Machekano, J. Madan, N. Beyers and P. Naidoo (2015). "Comparing multidrug-resistant tuberculosis patient costs under molecular diagnostic algorithms in South Africa." The International Journal of Tuberculosis and Lung Disease **19**(8): 960-968.

Dunbar, R., R. Van Hest, K. Lawrence, S. Verver, D. A. Enarson, C. Lombard, N. Beyers and J. M. Barnes (2011). "Capture-recapture to estimate completeness of tuberculosis surveillance in two communities in South Africa." The International Journal of Tuberculosis and Lung Disease **15**(8): 1038-1043.

Edginton, M. E., M. L. Wong, R. Phofa, D. Mahlaba and H. J. Hodkinson (2005). "Tuberculosis at Chris Hani Baragwanath Hospital: numbers of patients diagnosed and outcomes of referrals to district clinics." The International Journal of Tuberculosis and Lung Disease **9**(4): 398-402.

Edginton, M. E., M. L. Wong and H. J. Hodkinson (2006). "Tuberculosis at Chris Hani Baragwanath hospital: an intervention to improve patient referrals to district clinics." The International Journal of Tuberculosis and Lung Disease **10**(9): 1018-1022.

Elliott, E., H. R. Draper, P. Baitsiwe and M. M. Claassens (2014). "Factors affecting treatment outcomes in drug-resistant tuberculosis cases in the Northern Cape, South Africa." Public health action **4**(3): 201.

Foster, N., A. Vassall, S. Cleary, L. Cunnama, G. Churchyard and E. Sinanovic (2015). "The economic burden of TB diagnosis and treatment in South Africa." Social Science & Medicine **130**: 42-50.

Grossman, M. (1972a). "On the Concept of Health Capital and the Demand for Health." Journal of Political Economy **80 2**: 223-255.

Grossman, M. (1972b). The Demand for Health: A Theoretical and Empirical Investigation. New York, NBER.

Harries, A. D., I. D. Rusen, C.-Y. Chiang, S. G. Hinderaker and D. A. Enarson (2009). "Registering initial defaulters and reporting on their treatment outcomes [Unresolved issues]." The international journal of tuberculosis and lung disease **13**(7): 801-803.

Hou, Z., E. Van de Poel, E. Van Doorslaer, B. Yu and Q. Meng (2014). "Effects of NCMS on access to care and financial protection in China." Health Econ **23**(8): 917-934.

Ismail, N. A., H. M. Said, Z. Pinini, S. V. Omar, N. Beyers and P. Naidoo (2015). "Optimizing Mycobacterial Culture in Smear-Negative, Human Immunodeficiency Virus-Infected Tuberculosis Cases." PloS one **10**(11): e0141851.

Kakwani, N., A. Wagstaff and E. Van Doorslaer (1997). "Socioeconomic inequalities in health: Measurement, computation and statistical inference." Journal of Econometrics **77**(1): 87-104.

KNCV Tuberculosis Foundation (2012). Guidelines to measure the prevalence of active TB disease among health care workers.

Laibson, D. and J. A. List (2015). "Principles of (Behavioral) Economics." American Economic Review **105 5**: 385-390.

Ledibane, T. D., S. C. Motlhanke, A. Rose, W. H. Kruger, N. R. T. Ledibane and M. M. Claassens (2015). "Antiretroviral treatment among co-infected tuberculosis patients in integrated and non-integrated facilities." Public health action **5**(2): 112.

Lester, R. T., P. Ritvo, E. J. Mills, A. Kariri, S. Karanja, M. H. Chung, W. Jack, J. Habyarimana, M. Sadatsafavi, M. Najafzadeh, C. A. Marra, B. Estambale, E. Ngugi, T. B. Ball, L. Thabane, L. J. Gelmon, J. Kimani, M. Ackers and F. A. Plummer (2010). "Effects of a mobile phone short message service on antiretroviral treatment adherence in Kenya (WelTel Kenya1): a randomised trial." Lancet **376**(9755): 1838-1845.

Limwattananon, S., S. Neelsen, O. O'Donnell, P. Prakongsai, V. Tangcharoensathien and E. van Doorslaer (2015). "Universal Coverage with Supply-Side Reform: The Impact on Medical Expenditure Risk and Utilization in Thailand." Journal of Public Economics **121**: 79-94.

Loveday, M. and L. Vanleeuw (2015). Tuberculosis. District Health Barometer 2014/15. Durban, South Africa, Health Systems Trust.

Lutge, E., S. Lewin, J. Volmink, I. Friedman and C. Lombard (2013). "Economic support to improve tuberculosis treatment outcomes in South Africa: a pragmatic cluster-randomized controlled trial." Trials **14**: 154.

Lutge, E. E., C. S. Wiysonge, S. E. Knight, D. Sinclair and J. Volmink (2015). "Incentives and enablers to improve adherence in tuberculosis." Cochrane Database Syst Rev **9**: CD007952.

MacPherson, P., R. M. G. J. Houben, J. R. Glynn, E. L. Corbett and K. Kranzer (2014). "Pre-treatment loss to follow-up in tuberculosis patients in low-and lower-middle-income countries and high-burden countries: a systematic review and meta-analysis." Bulletin of the World Health Organization **92**(2): 126-138.

Marteau, T. M., D. Ogilvie, M. Roland, M. Suhrcke and M. P. Kelly (2011). "Judging nudging: can nudging improve population health?" BMJ **342**: d228.

Meehan, S.-A., P. Naidoo, M. M. Claassens, C. Lombard and N. Beyers (2014). "Characteristics of clients who access mobile compared to clinic HIV counselling and testing services: a matched study from Cape Town, South Africa." BMC health services research **14**(1): 1.

Meehan, S.-A., N. Leon, P. Naidoo, K. Jennings, R. Burger and N. Beyers (2015). "Availability and acceptability of HIV counselling and testing services. A qualitative study comparing clients' experiences of accessing HIV testing at public sector primary health care facilities or non-governmental mobile services in Cape Town, South Africa." BMC public health **15**(1): 845.

Migliori, G. B., A. Spanevello, L. Ballardini, M. Neri, C. Gambarini, M. L. Moro, L. Trnka and M. C. Raviglione (1995). "Validation of the surveillance system for new cases of tuberculosis in a province of northern Italy. Varese Tuberculosis Study Group." European respiratory journal **8**(8): 1252-1258.

Mngomezulu, N., D. Cameron, S. Olorunju, T. Luthuli, R. Dunbar and P. Naidoo (2015). "Reasons for the low bacteriological coverage of tuberculosis reported in Mpumalanga Province, South Africa." Public health action **5**(2): 122.

Moreno-Serra, R. and A. Wagstaff (2010). "System-wide impacts of hospital payment reforms: Evidence from Central and Eastern Europe and Central Asia." Journal of Health Economics **29**(4): 585-602.

Munir, M. K., R. Iqbal, I. Shabbir and K. Chaudhry (2012). "Factors Responsible for Failure to Initiate Tuberculosis Treatment among Smear Positive Tuberculosis Patients." Pakistan Journal of Medical Research **51**(2): 34.

Munro, S. A., S. A. Lewin, H. J. Smith, M. E. Engel, A. Fretheim and J. Volmink (2007). "Patient adherence to tuberculosis treatment: a systematic review of qualitative research." PLoS Med **4**(7): e238.

Murray, E. J., V. A. Bond, B. J. Marais, P. Godfrey-Faussett, H. M. Ayles and N. Beyers (2012). "High levels of vulnerability and anticipated stigma reduce the impetus for tuberculosis diagnosis in Cape Town, South Africa." Health policy and planning: czs072.

Murray, E. J., V. A. Bond, B. J. Marais, P. Godfrey-Faussett, H. M. Ayles and N. Beyers (2013). "High Levels of Vulnerability and Anticipated Stigma Reduce the Impetus for Tuberculosis Diagnosis in Cape Town, South Africa." Health Policy and Planning **28 4**: 410-418.

Naidoo, P., E. du Toit, R. Dunbar, C. Lombard, J. Caldwell, A. Detjen, S. B. Squire, D. A. Enarson and N. Beyers (2014). "A comparison of multidrug-resistant tuberculosis treatment commencement times in MDRTBPlus line probe assay and Xpert® MTB/RIF-based algorithms in a routine operational setting in Cape Town." PloS one **9**(7): e103328.

Naidoo, P., M. van Niekerk, E. du Toit, N. Beyers and N. Leon (2015). "Pathways to multidrug-resistant tuberculosis diagnosis and treatment initiation: a qualitative comparison of patients' experiences in the era of rapid molecular diagnostic tests." BMC health services research **15**(1): 1.

Naidoo, P., R. Dunbar, C. Lombard, E. du Toit, J. Caldwell, A. Detjen, S. B. Squire, D. A. Enarson and N. Beyers (2016). "Comparing Tuberculosis Diagnostic Yield in Smear/Culture and Xpert® MTB/RIF-Based Algorithms Using a Non-Randomised Stepped-Wedge Design." PloS one **11**(3): e0150487.

National Department of Health South Africa (2014). National Tuberculosis Management Guidelines 2014. Pretoria, South Africa.

Nguyen, H. T., S. Bales, A. Wagstaff and H. Dao (2015). "Getting Incentives Right? The Impact of Hospital Capitation Payment in Vietnam." Health Econ.

O'Donnell, O., E. van Doorslaer, A. Wagstaff and M. Lindelow (2008). Analyzing Health Equity Using Household Survey Data: A Guide to Techniques and Their Implementation. Washington DC, World Bank.

O'Donnell, O., E. van Doorslaer, A. Wagstaff and A. M. Jones (2012). Decomposition of Inequalities in Health and Health Care. The Elgar Companion to Health Economics, Second Edition. Cheltenham, U.K. and Northampton, Mass., Elgar**:** 179-191.

Palanivel, C., A. M. V. Kumar, T. Mahalakshmi, S. Govindarajan, M. Claassens, S. Satyanarayana, D. Gurumurthy, K. Vasudevan, A. Purty, A. K. Paulraj and others (2013). "Uptake of HIV testing and HIV positivity among presumptive tuberculosis patients at Puducherry, South India." Public health action **3**(3): 220.

Pilote, L., J. P. Tulsky, A. R. Zolopa, J. A. Hahn, G. F. Schecter and A. R. Moss (1996). "Tuberculosis prophylaxis in the homeless. A trial to improve adherence to referral." Arch Intern Med **156**(2): 161-165.

Podewils, L. J., N. Bantubani, C. Bristow, L. E. Bronner, A. Peters, A. Pym and L. D. Mametja (2015). "Completeness and Reliability of the Republic of South Africa National Tuberculosis (TB) Surveillance System." BMC public health **15**(1): 1.

Pop-Eleches, C., H. Thirumurthy, J. P. Habyarimana, J. G. Zivin, M. P. Goldstein, D. de Walque, L. MacKeen, J. Haberer, S. Kimaiyo, J. Sidle, D. Ngare and D. R. Bangsberg (2011). "Mobile phone technologies improve adherence to antiretroviral treatment in a resource-limited setting: a randomized controlled trial of text message reminders." AIDS **25**(6): 825-834.

Rao, M., A. Katyal, P. V. Singh, A. Samarth, S. Bergkvist, M. Kancharla, A. Wagstaff, G. Netuveli and A. Renton (2014). "Changes in addressing inequalities in access to hospital care in Andhra Pradesh and Maharashtra states of India: a difference-in-differences study using repeated cross-sectional surveys." BMJ Open **4**(6).

Raviglione, M. C. and M. W. Uplekar (2006). "WHO's new Stop TB Strategy." The Lancet **367**(9514): 952-955.

Sai Babu, B., A. V. V. Satyanarayana, G. Venkateshwaralu, U. Ramakrishna, P. Vikram, S. Sahu, F. Wares, P. K. Dewan, K. Santosha, J. Jyoti and others (2008). "Initial default among diagnosed sputum smear-positive pulmonary tuberculosis patients in Andhra Pradesh, India." The International Journal of Tuberculosis and Lung Disease **12**(9): 1055-1058.

Santha, T., G. Renu, T. R. Frieden, R. Subramani, P. G. Gopi, V. Chandrasekaran, N. Selvakumar, A. Thomas, R. Rajeswari, R. Balasubramanian and others (2003). "Are community surveys to detect tuberculosis in high prevalence areas useful? Results of a comparative study from Tiruvallur District, South India." The International Journal of Tuberculosis and Lung Disease **7**(3): 258-265.

Schulz, S. A., H. R. Draper and P. Naidoo (2013). "A comparative study of tuberculosis patients initiated on ART and receiving different models of TB-HIV care." The International Journal of Tuberculosis and Lung Disease **17**(12): 1558-1563.

Sinanovic, E., K. Floyd, L. Dudley, V. Azevedo, R. Grant and D. Maher (2003). "Cost and cost-effectiveness of community-based care for tuberculosis in Cape Town, South Africa." The international journal of tuberculosis and lung disease **7**(9s1): S56-S62.

Skinner, D. and M. M. Claassens (2016). It's complicated: why do tuberculosis patients not initiate treatment? A qualitative study from South Africa.

Squire, S. B., A. K. Belaye, A. Kashoti, F. M. L. Salaniponi, C. J. F. Mundy, S. Theobald and J. Kemp (2005). "'Lost'smear-positive pulmonary tuberculosis cases: where are they and why did we lose them?" The International Journal of Tuberculosis and Lung Disease **9**(1): 25-31.

Statistics South Africa (2015). Mortality and causes of death in South Africa, 2013: Findings from death notification, Statistics South Africa.

Styblo, K. and J. R. Bumgarner (1991). "Tuberculosis can be controlled with existing technologies: evidence." The Hague: Tuberculosis Surveillance Research Unit: 60-72.

Sun, X., X. Liu, Q. Sun, W. Yip, A. Wagstaff and Q. Meng (2016). "The Impact of a Pay-for-Performance Scheme on Prescription Quality in Rural China." Health Econ.

Thaler, R. H. and C. R. Sunstein (2008). Nudge: Improving Decisions about Health, Wealth, and Happiness. New Haven and London, Yale University Press.

Ukwaja, K. N., O. Modebe, C. Igwenyi and I. Alobu (2012). "The economic burden of tuberculosis care for patients and households in Africa: a systematic review [Review article]." The International Journal of Tuberculosis and Lung Disease **16**(6): 733-739.

Van de Poel, E., G. Flores, P. Ir, O. O'Donnell and E. Van Doorslaer (2014). "Can vouchers deliver? An evaluation of subsidies for maternal health care in Cambodia." Bull World Health Organ **92**(5): 331-339.

van Doorslaer, E., A. Wagstaff, H. Bleichrodt, S. Calonge, U. G. Gerdtham, M. Gerfin, J. Geurts, L. Gross, U. Hakkinen, R. E. Leu, O. ODonnell, C. Propper, F. Puffer, M. Rodriguez, G. Sundberg and O. Winkelhake (1997). "Income-related inequalities in health: Some international comparisons." Journal of Health Economics **16**(1): 93-112.

Van Doorslaer, E. K. (1987). Health, knowledge and the demand for medical care: An econometric analysis, van Gorcum Maastricht/Wolfeboro, Hampshire.

Victora, C. G., A. Wagstaff, J. A. Schellenberg, D. Gwatkin, M. Claeson and J. P. Habicht (2003). "Applying an equity lens to child health and mortality: more of the same is not enough." Lancet **362**(9379): 233-241.

Wagstaff, A. (1986). "The demand for health: Some new empirical evidence." Journal of Health Economics **5**(3): 195-233.

Wagstaff, A. (1993). "The demand for health: an empirical reformulation of the Grossman model." Health Econ **2**(2): 189-198.

Wagstaff, A. and E. van Doorslaer (2000). "Measuring and testing for inequity in the delivery of health care." Journal of Human Resources **35**(4): 716-733.

Wagstaff, A., E. van Doorslaer and N. Watanabe (2003). "On decomposing the causes of health sector inequalities, with an application to malnutrition inequalities in Vietnam." Journal of Econometrics **112**(1): 207-223.

Wagstaff, A., F. Bustreo, J. Bryce, M. Claeson and W.-W. B. C. H. P. W (2004). "Child health: Reaching the poor." American Journal of Public Health **94**(5): 726-736.

Wagstaff, A. and M. Claeson (2004). The Millennium Development Goals For Health : Rising to the Challenges. Washington, DC, World Bank.

Wagstaff, A., M. Lindelow, G. Jun, X. Ling and Q. Juncheng (2009). "Extending Health Insurance to the Rural Population: An Impact Evaluation of China's New Cooperative Medical Scheme." Journal of Health Economics **28 1**: 1-19.

Wagstaff, A. and R. Moreno-Serra (2009). "Europe and central Asia's great post-communist social health insurance experiment: Aggregate impacts on health sector outcomes." Journal of Health Economics **28**(2): 322-340.

Wagstaff, A. (2010). "Estimating Health Insurance Impacts under Unobserved Heterogeneity: The Case of Vietnam's Health Care Fund for the Poor." Health Economics **19**(2): 189-208.

Wagstaff, A., C. Bredenkamp and L. R. Buisman (2014). "Progress on Global Health Goals: are the Poor Being Left Behind?" The World Bank Research Observer **29**(2): 137-162.

Wagstaff, A., H. T. Nguyen, H. Dao and S. Bales (2015). "Encouraging Health Insurance for the Informal Sector: A Cluster Randomized Experiment in Vietnam." Health Econ.

World Bank (2015). World Development Report 2015: Mind, Society, and Behavior, Washington and New York: International Bank for Reconstruction and Development/The World Bank.

World Health Organization (1994). WHO Tuberculosis Programme : framework for effective tuberculosis control. Geneva, Switzerland: 1-13.

World Health Organization (2015). Global tuberculosis report 2015. Geneva, Switzerland: 1-204.

World Health Organization. (2016, 2016/04/14/). "Tuberculosis profile of South Africa: Population 2014." from https://extranet.who.int/sree/Reports?op=Replet&name=%2FWHO_HQ_Reports%2FG2%2FPROD%2FEXT%2FTBCountryProfile&ISO2=ZA&LAN=EN&outtype=html.

Zhang, H., T. Bago d'uva and E. van Doorslaer (2015). "The Gender Health Gap in China: A Decomposition Analysis." Economics and Human Biology **18**: 13-26.

Annexure A1

UNIVERSITEIT•STELLENBOSCH•UNIVERSITY
jou kennisvennoot • your knowledge partner

# Study: Social and Psychological/Behavioral Factors Influencing Non-Initiation of TB Treatment

## Retrospective Patient Journeys

---

Study: Evaluation of molecular diagnostic tests for TB (IRB0005239)(N10/09/308)

**INFORMATION LEAFLET**

PRINCIPAL INVESTIGATOR: Dr Pren Naidoo
ADDRESS: Desmond Tutu TB Centre, Faculty of Health Sciences, Stellenbosch University, Francie van Zyl Avenue, Parow
CONTACT NUMBER: 021 938 9114

STUDY PRINCIPLE INVESTIGATOR: Dr Ronelle Burger
Department of Economics, Stellenbosch University
CONTACT NUMBER: 021 808 3106

You are being invited to take part in a research study. Please take some time to read this information, which will explain the details of the study. Please ask the researchers any questions about the study that you do not fully understand. It is important that you understand what this research involves. Your participation is entirely voluntary. You are free to choose NOT to participate. If you say no, this will not affect you negatively in any way whatsoever. If you agree to participate, you are also free to withdraw from the study at any point.

This study has been approved by the Health Research Ethics Committee: Humanities (HREC) at Stellenbosch University and will be conducted according to national and international ethical guidelines and principles.

**What is this research study all about?**

- We want to know more about what people think about TB and how they make decisions about coming to the clinic for tests and treatment. This will help us find ways to make it easier for people to get TB treatment.
- You have been chosen to take part in the study because you have recently completed your TB treatment journey
- This study is taking place at 10 clinics in Cape Town. All people over 18 years of age who come for a TB test at these clinics will be asked if they are willing to take part in the study.

**What will your responsibilities be?**

- You will be asked to complete a questionnaire. A researcher will be available to help you to do this or to complete it for you if you are unable to.be asked some questions and will be given the chance to talk to a researcher about your experience with TB and its treatment. Please answer these questions honestly and as best you can.
- The questionnaire interview should take about an hour of your time.

- We ask you to try to answer the questions as accurately as possible.
- ~~We ask you to wear a face mask to avoid the spread of germs.~~

**Will you benefit from taking part in this research?**

- There are no personal benefits for taking part in this study. We hope to find ways to make it easier for people to get tested and start TB treatment in the future.

**Are there in risks involved in your taking part in this research?**

- We do not foresee any risks in your taking part in the study. You are free to refuse to answer any questions that you do not want to answer or to withdraw from the study at any time.
- If you want to talk to someone about your TB experience, there are counselors available at the clinics that are available to help you. Their contact details are at the bottom of this information sheet.

**What will happen if you do not agree to take part in the study?**

- You will still receive ALL the care and treatment you need if you do NOT agree to take part in the study.

**How will your information be used?**

- Your information is confidential and only the researchers will have access to your personal information.
- All reports from the study will only show summary information for example, how many men and women were interviewed, how many were employed, how many live in brick houses etc.
- We will not use your name in any reports. This will prevent anyone being able to identify you.

**Will you be paid to take part in this study and are there any costs involved?**

- You will NOT be paid to take part in the study. There will be no costs involved for you, if you do take part.

**Do you have any questions about the research?**

- If there is anything else that you want to know later, or if you have any problems related to your participation in this study you can contact Dr Ronelle Burger (Telephone number: 021 808 3106).
- If you have any questions about your rights as a participant, contact Maléne Fouché [mfouche@sun.ac.za or 021 808 4622] also at Stellenbosch University.

~~**You can contact the Health Research Ethics Committee at 021-938 9207 if you have any concerns or complaints that have not been addressed by the researcher.**~~

**Counsellors Contact details:**

*Will be added when the Western Cape Department of Health has granted permission for the study. This is to ensure up-to-date details.*

**You will receive a copy of this information leaflet for your own records.**

UNIVERSITEIT•STELLENBOSCH•UNIVERSITY
jou kennisvennoot • your knowledge partner

## Study: Social and Psychological/Behavioral Factors Influencing Non-Initiation of TB Treatment

## Retrospective Patient Journeys

~~Study: Evaluation of molecular diagnostic tests for TB (IRB0005239)(N10/09/308)~~

**CONSENT TO BE INTERVIEWED AND TAKE PART IN STUDY**

**PARTCIPANT**

**I,__________________________________________________ (Name) voluntarily agree to participate in this study.** I agree that:

- I have read or had read to me this information and consent form and it is written in a language that I understand.
- I have had a chance to ask questions and all my questions have been answered.
- I understand that it is my choice to answer these questions and take part in this study.
- I may choose to leave or stop answering questions at any time and not be part of the study. This will not affect the care that I receive in any way.

***SIGNATURE OF PARTICIPANT***

Signed at (*place*) __________________________________on (*date*)____________________________

________________________________________ ________________________________________

Signature of participant Signature of witness

**INVESTIGATOR**

**I,__________________________________________________ (Name)** declare that I have explained the information in this document to the participant.

- I encouraged him/her to ask questions and took adequate time to answer them.
- I am satisfied that he/she adequately understands the information provided

***SIGNATURE OF INTERVIEWER***

____________________________________ ______________________

Signature of Interviewer Date

Annexure A2

UNIVERSITEIT•STELLENBOSCH•UNIVERSITY
jou kennisvennoot • your knowledge partner

# Study: Social and Psychological/Behavioral Factors Influencing Non-Initiation of TB Treatment

## Waiting Room Surveys

---

### INFORMATION LEAFLET

PRINCIPLE INVESTIGATOR: Dr Ronelle Burger
Department of Economics, Stellenbosch University
CONTACT NUMBER: 021 808 3106

You are being invited to take part in a research study. Please take some time to read this information, which will explain the details of the study. Please ask the researchers any questions about the study that you do not fully understand. It is important that you understand what this research involves. Your participation is entirely voluntary. You are free to choose NOT to participate. If you say no, this will not affect you negatively in any way whatsoever. If you agree to participate, you are also free to withdraw from the study at any point.

This study has been approved by the Research Ethics Committee: Humanities (REC) at Stellenbosch University and will be conducted according to national and international ethical guidelines and principles.

**What is this research study all about?**

- We want to know more about what people think about TB and how they make decisions about coming to the clinic for tests and treatment. This will help us find ways to make it easier for people to get TB treatment.
- You have been chosen to take part in the study because you have come to the clinic and are being tested for TB.
- Please note that the researcher is wearing a mask for their own protection.

**What will your responsibilities be?**

- You will be be asked to fill out a survey about your understanding of TB and TB treatment. Please answer these questions honestly and as best you can.
- The survey will not take more than an hour of your time.
- We ask you to try to answer the questions as accurately as possible.

**Will you benefit from taking part in this research?**

- There are no personal benefits for taking part in this study. We hope to find ways to make it easier for people to get tested and start TB treatment in the future.

**Are there in risks involved in your taking part in this research?**

- We do not foresee any risks in your taking part in the study. You are free to refuse to answer any questions that you do not want to answer or to withdraw from the study at any time.
- If you want to talk to someone about your TB experience, there are counselors available at the clinic that are available to help you. Their contact details are at the bottom of this information sheet.

**What will happen if you do not agree to take part in the study?**

- You will still receive ALL the care and treatment you need if you do NOT agree to take part in the study.

**How will your information be used?**

- Your information is confidential and only the researchers will have access to your personal information.
- All reports from the study will only show summary information for example, how many men and women were interviewed, how many were employed, how many live in brick houses etc.
- We will not use your name in any reports. This will prevent anyone being able to identify you.

**Will you be paid to take part in this study and are there any costs involved?**

- You will NOT be paid to take part in the study. There will be no costs involved for you, if you do take part.

**Do you have any questions about the research?**

- If there is anything else that you want to know later, or if you have any problems related to your participation in this study you can contact Dr Ronelle Burger (Telephone number: 021 808 3106).
- If you have any questions about your rights as a participant, contact Maléne Fouché [mfouche@sun.ac.za or 021 808 4622] also at Stellenbosch University.

**Counsellors Contact details:**

*Will be added when the Western Cape Department of Health has granted permission for the study. This is to ensure up-to-date details.*

**You will receive a copy of this information leaflet for your own records.**

UNIVERSITEIT•STELLENBOSCH•UNIVERSITY
jou kennisvennoot • your knowledge partner

# Study: Social and Psychological/Behavioral Factors Influencing Non-Initiation of TB Treatment

## Waiting Room Surveys

### CONSENT TO BE INTERVIEWED AND TAKE PART IN STUDY

**PARTCIPANT**

**I,____________________________________________ (Name) voluntarily agree to participate in this study.** I agree that:

- I have read or had read to me this information and consent form and it is written in a language that I understand.
- I have had a chance to ask questions and all my questions have been answered.
- I understand that it is my choice to answer these questions and take part in this study.
- I may choose to leave or stop answering questions at any time and not be part of the study. This will not affect the care that I receive in any way.

***SIGNATURE OF PARTICIPANT***

Signed at (*place*) ______________________________ on (*date*)_________________________

____________________________________ ____________________________________

Signature of participant Signature of witness

**INVESTIGATOR**

**I,____________________________________________ (Name)** declare that I have explained the information in this document to the participant.

- I encouraged him/her to ask questions and took adequate time to answer them.
- I am satisfied that he/she adequately understands the information provided

***SIGNATURE OF INTERVIEWER***

Signature of Interviewer Date

## ANNEXURE B: QUESTIONNAIRE ABOUT YOUR HEALTH

| | |
|---|---|
| **Your name:** | |
| **Your clinic folder number:** | |
| **The address where you now live:** | |

## TICK THE CORRECT BOX/BOXES

### SECTION 1 – MAKING CHOICES

| | | | |
|---|---|---|---|
| 1. Would you prefer to receive R200 guaranteed *today* or R250 guaranteed in *one month from today* (calculate and provide date)? | Today | 1 Month | → If "R250 One month" skip next question |
| 2. Would you prefer to receive R200 guaranteed *today* or R300 guaranteed in *one month from today* (calculate and provide date)? | Today | 1 Month | |
| 3. Which guaranteed amount would you like to receive in *one month from today* (calculate and provide date) that will make you just as happy as R200 *today*? | | | |
| 4. Would you prefer to receive R200 guaranteed *six months from today* or R250 guaranteed in *seven months from today* (calculate and provide date)? | 6 Months | 7 Months | → If "R250 Seven months" skip next question |
| 5. Would you prefer to receive R200 guaranteed *six months from today* or R300 guaranteed in *seven months from today* (calculate and provide date)? | 6 Months | 7 Months | |
| 6. Which guaranteed amount would you like to receive in *seven months from today* (calculate and provide date) that will make you just as happy as R200 *six months from today*? | | | |

7. Imagine the following situation: you are buying groceries and the total comes to R100. While you are standing in line, you see that the store offers a R10 discount if you pay in cash so you decide to pay cash:

   You look in your wallet and see that you have the cash so you will get the R10 discount. How would you feel about getting the R10 discount?

| 1. Very Unhappy | 2. Unhappy | 3. Neutral | 4. Happy | 5. Very Happy |
|---|---|---|---|---|
| | | | | |

8. Imagine the following situation: you are buying groceries and the total comes to R100. While you are standing in line, you see that the store offers a R10 discount if you pay in cash so you decide to pay cash:

   You look in your wallet and see that you don't have the cash, so you will not get the discount. How would you feel using your bank card (debit card) and not getting the R10 discount?

| 1. Very Unhappy | 2. Unhappy | 3. Neutral | 4. Happy | 5. Very Happy |
|---|---|---|---|---|

| | | | | |
|---|---|---|---|---|
| | | | | |

9. Imagine the following situation: you are buying groceries and the total comes to R100. While you are standing in line, you see that the store charges a R10 extra charge for paying with your card and you decide to pay cash:

You look in your wallet and see that you have cash so you will not have to pay the extra charge. How would you feel paying in cash and not paying the R10 extra charges?

| 1. Very Unhappy | 2. Unhappy | 3. Neutral | 4. Happy | 5. Very Happy |
|---|---|---|---|---|
| | | | | |

10. Imagine the following situation: you are buying groceries and the total comes to R100. While you are standing in line, you see that the store charges a R10 extra charge for paying with your card and you decide to pay cash:

You look in your wallet and see that you don't have the cash, so you will be paying the extra charge. How would you feel about using your card and paying the R10 extra charges?

| 1. Very Unhappy | 1. Very Unhappy | 1. Very Unhappy | 1. Very Unhappy | 1. Very Unhappy |
|---|---|---|---|---|
| | | | | |

## SECTION 2 – GENERAL HEALTH AND SYMPTOMS

| | M | M | Y | Y | Y | Y |
|---|---|---|---|---|---|---|
| 1. When was the last time you felt really healthy? | | | | | | |

| | Yes | No |
|---|---|---|
| 2. Are you currently feeling unwell? | | |

| | M | M | Y | Y | Y | Y |
|---|---|---|---|---|---|---|
| 3. If yes, when did you start to feel unwell? | | | | | | |

4. If you are feeling unwell, did you experience any of the symptom/s below? Please tick all that apply

| Persistent cough | Cough with blood | Fever | Night sweats | Chest pain | Generally not feeling well | Weight loss | Other |
|---|---|---|---|---|---|---|---|
| | | | | | | | |

5. What symptoms are bothering you the most? Please tick all that apply

| Persistent cough | Cough with blood | Fever | Night sweats | Chest pain | Generally not feeling well | Weight loss | Other |
|---|---|---|---|---|---|---|---|

| | | | | | | | |
|---|---|---|---|---|---|---|---|
| | | | | | | | |

| | Yes | No |
|---|---|---|
| 6. If you are currently feeling unwell, did you seek medical care before today? | | |

7. Since you started feeling unwell, please indicate where you have gone for help and how many times you visited each place (you can select more than one)

| Pharmacy | GP | Public Clinic | Hospital | Traditional healer | Other *Provide details below please* |
|---|---|---|---|---|---|
| | | | | | |

8. What motivated you to seek care today? Please tick all that apply to this visit

| Felt very ill | Family said I should come | Friends said I should come | Nurse or doctor said I should come | Someone else said I should come | Came to clinic for other reason | Worried that I might have TB | Other *Provide details below please* |
|---|---|---|---|---|---|---|---|
| | | | | | | | |

| 9. In the last week, what have you been worried about? | Tick all that apply | Rank worries from 1 to 10 (1 = most worried; 8 = least worried) |
|---|---|---|
| Feeding my family | | |
| My health | | |
| Being able to get to/from work | | |
| Children in my household being able to go to school | | |
| The health of children in my household or other family members | | |
| Finding work/ keeping my job | | |
| Going hungry | | |
| My relationship with my spouse or other family members | | |

10. How important are health concerns to you?

| 1. Very Important | 2. Somewhat important | 3. Neutral | 4. Not very important | 5. Not at all important |
|---|---|---|---|---|
| | | | | |

11. How important is it to you to see a nurse and get the pills you need if you are ill?

| 1. Very Important | 2. Somewhat important | 3. Neutral | 4. Not very important | 5. Not at all important |
|---|---|---|---|---|

| | | | | |
|---|---|---|---|---|
| | | | | |

## SECTION 3 – TUBERCULOSIS KNOWLEDGE AND BELIEFS

1. [Name] has been diagnosed with tuberculosis. Please indicate whether you agree or disagree with each of the statements below.

| | 1. Strongly agree | 2. Agree | 3. Neutral | 4. Disagree | 5. Strongly disagree |
|---|---|---|---|---|---|
| This will make it hard for [Name] to get a job | | | | | |
| I would be comfortable working with [Name] | | | | | |
| I would be comfortable having [Name] over for dinner | | | | | |
| It is likely that [Name}'s wife/husband will leave him/her | | | | | |

2. A child in [Name]'s house has been coughing and losing weight. Please indicate whether you agree or disagree with each of the statements below.

| | 1. Strongly agree | 2. Agree | 3. Neutral | 4. Disagree | 5. Strongly disagree |
|---|---|---|---|---|---|
| The child should go to the clinic | | | | | |
| The child will make others sick | | | | | |
| [Name] should wait and see if the symptoms get better before taking the child to the doctor/nurse | | | | | |

| 3. Please answer yes or no to the following statements | Yes | No |
|---|---|---|
| TB can be easily cured | | |
| I know a lot about TB and its treatment | | |
| All people with TB will develop HIV/AIDS | | |
| One only needs to worry about TB if you are coughing blood | | |
| Curing TB takes a long time | | |
| TB medication is expensive | | |
| I know someone who has had TB | | |
| I know someone who has died from TB | | |
| People who do not get treated for TB are irresponsible | | |
| If you have TB, people do not respect you | | |
| It is the people that do not get treatment for TB that make other people sick | | |
| I know someone who was cured of TB | | |
| If you had TB before, you cannot get it again | | |

| | | |
|---|---|---|
| TB treatment has bad side effects | | |
| You cannot work and be on TB treatment | | |
| TB can be cured, but the clinic treatment is not effective | | |
| Often people go on TB treatment, but are not cured | | |
| I think I might have TB | | |

4. How likely do you think it is that you have TB?

| 1. Very likely | 2. Somewhat likely | 3. Neutral | 4. Unlikely | 5. Extremely unlikely |
|---|---|---|---|---|
| | | | | |

5. If you close your eyes and imagine a typical TB patient, please tick all of the characteristics that apply to him/her. Is the person:

| | | | |
|---|---|---|---|
| Young | | Rich | |
| Clean | | Female | |
| Old | | Poor | |
| Dirty | | Male | |
| Smoking | | Thin | |
| Drinking | | HIV positive | |
| Unemployed | | Look and feel very sick | |

## SECTION 4 – TUBERCULOSIS DIAGNOSIS AND TREATMENT

| | Yes | No |
|---|---|---|
| 1. Have you been tested for TB before? | | |

| 2. If yes, please answer the following questions | Yes | No |
|---|---|---|
| Was it a positive experience? | | |
| Did you have to wait long? | | |
| Was the nurse friendly? | | |
| Did she explain to you that she is testing you for TB? | | |
| Did she explain to you why she is testing you for TB? | | |
| Did she ask you to come back? | | |
| Did she ask you to come back on a specific day? | | |

| | | |
|---|---|---|
| Did you go back to the clinic? | | |

| 3. If you did not go back to the clinic, why not? Please tick three reasons that apply and rank them in order of importance | Yes | No | Rank |
|---|---|---|---|
| I forgot to go back | | | |
| I am still planning to go back to clinic | | | |
| I did not feel ill | | | |
| I do not think I have TB | | | |
| I have been too busy | | | |
| I am afraid of getting my test results | | | |
| I don't like the clinic | | | |
| The nurses at the clinic are unfriendly | | | |
| I am worried about side effects of treatment | | | |
| I could not get time off work | | | |
| I went to the clinic, but they could not give me the results | | | |
| I don't have time for TB treatment | | | |
| I don't have the money for TB treatment | | | |
| I am ashamed | | | |
| There is no privacy at the clinic | | | |
| I did not know that I had to go back to the clinic | | | |
| I went elsewhere, for example traditional healer or private practice | | | |

4. How easy is it for you to come to the clinic?

| 1. Very easy | 2. Easy | 3. Neither easy nor difficult | 4. Not easy/difficult | 5. Very difficult |
|---|---|---|---|---|
| | | | | |

5. How well do you understand the TB treatment process?

| 1. Very well | 2. Well | 3. Understand some things | 4. Not well | 5. Not at all |
|---|---|---|---|---|
| | | | | |

| 6. If you have TB, you will need to (select those that you agree with): | | | |
|---|---|---|---|
| Attend the clinic every day for 6 months | | Attend the clinic every day 12 months | |
| Attend the clinic every day for 24 months | | Receive injections every day for 2 months | |
| Take treatment for 6 months at a place that is convenient for me | | Not take medication, because none is available | |

| | Yes | No |
|---|---|---|
| 7. Do you intend to return to the clinic to collect your results? | | |
| 8. When will you return to the clinic to collect your results (DD/MM/YYYY)? | | |

## SECTION 5 – SOCIO-ECONOMIC AND LIFESTYLE

1. What type of house do you live in?

| | |
|---|---|
| Brick house | 1 |
| Traditional dwelling/hut/structure | 2 |
| Informal dwelling or shack | 3 |
| Other | -8 |

| 2. In your household is there: | Yes | No |
|---|---|---|
| Someone with a university degree | | |
| Someone with a job | | |
| Electricity | | |
| Hot water running from a tap | | |
| Television | | |
| Refrigerator/freezer | | |
| Satellite dish | | |
| Car | | |
| Someone with a mobile phone | | |

| | Yes | No |
|---|---|---|
| 3. Do you use electricity for heating? | | |
| 4. Do you use electricity for cooking? | | |

| | Yes | No | Unknown |
|---|---|---|---|
| 5. During the last 4 weeks, have you taken any drinks containing alcohol? | 1 | 0 | -5 |
| 6. During the last year have you ever drunk so much that you were unable to remember what you were doing? | 1 | 0 | -5 |

| | | |
|---|---|---|
| 7. Please tell us about your smoking habits: | Daily smoker | 1 |
| | Occasional smoker | 2 |
| | Ex-smoker | 3 |
| | Never smoked | 4 |

| If smoker, occasional smoker or ex-smoker continue, if never-smoker skip to next section | | | |
|---|---|---|---|
| 8. How long have you/did you smoke for (Years) [999 if unknown] | | | |
| 9. How many cigarettes did/do you smoke per day? [999 if unknown] | | | |

| | Yes | No | Unknown |
|---|---|---|---|
| 10. Do you ever smoke marijuana/dagga? | | | |
| 11. Do you ever take other drugs such as cocaine, TIK or speed? | | | |

| PARTICIPANT FOLLOW-UP & CONTACT DETAILS | | |
|---|---|---|
| **Are willing to receive sms information from the study team?** | YES☐ | NO☐ |
| **Are willing to be contacted at a later stage to answer more questions?** | YES☐ | NO☐ |
| **If you are willing to receive a sms or to be contacted at a later stage to answer more questions** | | |
| Please provide your telephone or cell number: | | |
| Please provide the name and telephone or cell number of a family member who we can contact you through: | | |
| Please provide the name or telephone or cell number of a friend who we can contact you through: | | |

| TO BE COMPLETED BY FIELD RESEARCHER | |
|---|---|
| Date of Interview | ____/______/______ (DD/MM/YYYY) |
| Researcher's Name | |
| Start Time - End time | _______________ to ______________ (eg 11:30 to 12:15) |
| Laboratory test results | Gene Xperrt Pos☐ Neg☐ Date received____/______/______ (DD/MM/YYYY) |
| | Smear result Pos☐ Neg☐ Date received____/______/______ (DD/MM/YYYY) |
| | Culture result Pos☐ Neg☐ Date received____/______/______ (DD/MM/YYYY) |
| Date patient first returned to facility | ____/______/______ (DD/MM/YYYY) Result provided YES☐ NO☐ |
| Reason if result not provided | |

# Appendix 2

**EForm Name**: REC: Humanities New Application
**Page**: Page 7
**Section**: 9.1 Consent form(s) (Please click on the yellow "+" in the right hand corner to add)
**Question**: Upload consent form(s)
**File Name**: icf_and_info_patient_161019.docx

UNIVERSITEIT·STELLENBOSCH·UNIVERSITY
jou kennisvennoot • your knowledge partner

**Study: Social and Psychological/Behavioral Factors Influencing Non-Initiation of TB Treatment**

**Retrospective Patient Journeys**

---

**INFORMATION LEAFLET**

PRINCIPLE INVESTIGATOR: Dr Ronelle Burger

Department of Economics, Stellenbosch University

CONTACT NUMBER: 021 808 3106

You are being invited to take part in a research study. Please take some time to read this information, which will explain the details of the study. Please ask the researchers any questions about the study that you do not fully understand. It is important that you understand what this research involves. Your participation is entirely voluntary. You are free to choose NOT to participate. If you say no, this will not affect you negatively in any way whatsoever. If you agree to participate, you are also free to withdraw from the study at any point.

This study has been approved by the Research Ethics Committee: Humanities (REC) at Stellenbosch University and will be conducted according to national and international ethical guidelines and principles.

**What is this research study all about?**

- We want to know more about what people think about TB and how they make decisions about coming to the clinic for tests and treatment. This will help us find ways to make it easier for people to get TB treatment.
- You have been chosen to take part in the study because you have recently completed your TB treatment journey
- .

**What will your responsibilities be?**

- You will be be asked some questions and will be given the chance to talk to a researcher about your experience with TB and its treatment. Please answer these questions honestly and as best you can.
- The interview should take about an hour of your time.
- We ask you to try to answer the questions as accurately as possible.
-

**Will you benefit from taking part in this research?**

- There are no personal benefits for taking part in this study. We hope to find ways to make it easier for people to get tested and start TB treatment in the future.

**Are there in risks involved in your taking part in this research?**

- We do not foresee any risks in your taking part in the study. You are free to refuse to answer any questions that you do not want to answer or to withdraw from the study at any time.

- If you want to talk to someone about your TB experience, there are counselors available at the clinics that are available to help you. Their contact details are at the bottom of this information sheet.

**What will happen if you do not agree to take part in the study?**

- You will still receive ALL the care and treatment you need if you do NOT agree to take part in the study.

**How will your information be used?**

- Your information is confidential and only the researchers will have access to your personal information.
- All reports from the study will only show summary information for example, how many men and women were interviewed, how many were employed, how many live in brick houses etc.
- We will not use your name in any reports. This will prevent anyone being able to identify you.

**Will you be paid to take part in this study and are there any costs involved?**

- You will NOT be paid to take part in the study. There will be no costs involved for you, if you do take part.

**Do you have any questions about the research?**

- If there is anything else that you want to know later, or if you have any problems related to your participation in this study you can contact Dr Ronelle Burger (Telephone number: 021 808 3106).
- If you have any questions about your rights as a participant, contact Maléne Fouché [mfouche@sun.ac.za or 021 808 4622] also at Stellenbosch University.

**Counsellors Contact details:**

*Will be added when the Western Cape Department of Health has granted permission for the study. This is to ensure up-to-date details.*

**You will receive a copy of this information leaflet for your own records.**

UNIVERSITEIT•STELLENBOSCH•UNIVERSITY
jou kennisvennoot • your knowledge partner

## Study: Social and Psychological/Behavioral Factors Influencing Non-Initiation of TB Treatment

## Retrospective Patient Journeys

---

**CONSENT TO BE INTERVIEWED AND TAKE PART IN STUDY**

**PARTCIPANT**

**I,_______________________________________________________________ (Name) voluntarily agree to participate in this study.** I agree that:

- I have read or had read to me this information and consent form and it is written in a language that I understand.
- I have had a chance to ask questions and all my questions have been answered.
- I understand that it is my choice to answer these questions and take part in this study.
- I may choose to leave or stop answering questions at any time and not be part of the study. This will not affect the care that I receive in any way.

***SIGNATURE OF PARTICIPANT***

Signed at (*place*) ____________________________________________ on (*date*)____________________________________

_____________________________________________ _________________________________________

Signature of participant Signature of witness

**INVESTIGATOR**

**I,_______________________________________________________________ (Name)** declare that I have explained the information in this document to the participant.

- I encouraged him/her to ask questions and took adequate time to answer them.
- I am satisfied that he/she adequately understands the information provided

***SIGNATURE OF INTERVIEWER***

________________________________________ _________________________

Signature of Interviewer Date

# Appendix 3

**EForm Name**: REC: Humanities New Application
**Page**: Page 7
**Section**: 9.1 Consent form(s) (Please click on the yellow "+" in the right hand corner to add)
**Question**: Upload consent form(s)
**File Name**: icf_and_info_waiting_room_161019.docx

UNIVERSITEIT•STELLENBOSCH•UNIVERSITY
jou kennisvennoot • your knowledge partner

## Study: Social and Psychological/Behavioral Factors Influencing Non-Initiation of TB Treatment

## Waiting Room Surveys

---

**INFORMATION LEAFLET**


PRINCIPLE INVESTIGATOR: Dr Ronelle Burger
Department of Economics, Stellenbosch University

CONTACT NUMBER: 021 808 3106


You are being invited to take part in a research study. Please take some time to read this information, which will explain the details of the study. Please ask the researchers any questions about the study that you do not fully understand. It is important that you understand what this research involves. Your participation is entirely voluntary. You are free to choose NOT to participate. If you say no, this will not affect you negatively in any way whatsoever. If you agree to participate, you are also free to withdraw from the study at any point.

This study has been approved by the Research Ethics Committee: Humanities (REC) at Stellenbosch University and will be conducted according to national and international ethical guidelines and principles.

**What is this research study all about?**

- We want to know more about what people think about TB and how they make decisions about coming to the clinic for tests and treatment. This will help us find ways to make it easier for people to get TB treatment.
- You have been chosen to take part in the study because you have come to the clinic and are being tested for TB.
- Please note that the researcher is wearing a mask for their own protection.

**What will your responsibilities be?**

- You will be be asked to fill out a survey about your understanding of TB and TB treatment. Please answer these questions honestly and as best you can.
- The survey will not take more than an hour of your time.
- We ask you to try to answer the questions as accurately as possible.

**Will you benefit from taking part in this research?**

- There are no personal benefits for taking part in this study. We hope to find ways to make it easier for people to get tested and start TB treatment in the future.

**Are there in risks involved in your taking part in this research?**

- We do not foresee any risks in your taking part in the study. You are free to refuse to answer any questions that you do not want to answer or to withdraw from the study at any time.
- If you want to talk to someone about your TB experience, there are counselors available at the clinic that are available to help you. Their contact details are at the bottom of this information sheet.

**What will happen if you do not agree to take part in the study?**

- You will still receive ALL the care and treatment you need if you do NOT agree to take part in the study.

**How will your information be used?**

- Your information is confidential and only the researchers will have access to your personal information.
- All reports from the study will only show summary information for example, how many men and women were interviewed, how many were employed, how many live in brick houses etc.
- We will not use your name in any reports. This will prevent anyone being able to identify you.

**Will you be paid to take part in this study and are there any costs involved?**

- You will NOT be paid to take part in the study. There will be no costs involved for you, if you do take part.

**Do you have any questions about the research?**

- If there is anything else that you want to know later, or if you have any problems related to your participation in this study you can contact Dr Ronelle Burger (Telephone number: 021 808 3106).
- If you have any questions about your rights as a participant, contact Maléne Fouché [mfouche@sun.ac.za or 021 808 4622] also at Stellenbosch University.

**Counsellors Contact details:**

*Will be added when the Western Cape Department of Health has granted permission for the study. This is to ensure up-to-date details.*

**You will receive a copy of this information leaflet for your own records.**

UNIVERSITEIT•STELLENBOSCH•UNIVERSITY
jou kennisvennoot • your knowledge partner

## Study: Social and Psychological/Behavioral Factors Influencing Non-Initiation of TB Treatment

## Waiting Room Surveys

**CONSENT TO BE INTERVIEWED AND TAKE PART IN STUDY**

**PARTCIPANT**

**I,_________________________________________________________________ (Name) voluntarily agree to participate in this study.** I agree that:

- I have read or had read to me this information and consent form and it is written in a language that I understand.
- I have had a chance to ask questions and all my questions have been answered.
- I understand that it is my choice to answer these questions and take part in this study.
- I may choose to leave or stop answering questions at any time and not be part of the study. This will not affect the care that I receive in any way.

***SIGNATURE OF PARTICIPANT***

Signed at (*place*) ____________________________________________on (*date*)____________________________________

__________________________________________ __________________________________________

Signature of participant Signature of witness

**INVESTIGATOR**

**I,_________________________________________________________________ (Name)** declare that I have explained the information in this document to the participant.

- I encouraged him/her to ask questions and took adequate time to answer them.
- I am satisfied that he/she adequately understands the information provided

***SIGNATURE OF INTERVIEWER***

________________________________________ _________________________

Signature of Interviewer Date

# Appendix 4

**EForm Name**: REC: Humanities New Application
**Page**: Page 9
**Section**: 12.2 Method of Data Collection
**Question**: Attach Here (if applicable)
**File Name**: patient_journeys20160905.docx

## RETROSPECTIVE PATIENT JOURNEY QUENSTIONNAIRE

| | |
|---|---|
| **Your name:** | |
| **I hereby consent voluntarily to be recorded during the completion of the survey (signature required):** | |
| **Have you completed treatment?** | |

## SECTION 1 – GENERAL HEALTH AND SYMPTOMS PRIOR TO BEING TESTED

| | D | D | M | M |
|---|---|---|---|---|
| 1. How long where did you feel ill for before you sought treatment? | | | | |

| | Yes | No |
|---|---|---|
| 2. Were you feeling unwell when you sought treatment? | | |

3. If you were feeling unwell, did you experience any of the symptom/s below? Please tick all that apply

| Persistent cough | Cough with blood | Fever | Night sweats | Chest pain | Generally not feeling well | Weight loss | Other |
|---|---|---|---|---|---|---|---|
| | | | | | | | |

4. What symptoms bothered you the most? Please tick all that apply

| Persistent cough | Cough with blood | Fever | Night sweats | Chest pain | Generally not feeling well | Weight loss | Other |
|---|---|---|---|---|---|---|---|
| | | | | | | | |

| | Yes | No |
|---|---|---|
| 5. Did you seek medical care before being tested for TB at the clinic? | | |

6. If yes, please indicate where you have gone for help and how many times you visited each place (you can select more than one)

| Pharmacy | GP | Public Clinic | Hospital | Traditional healer | Other<br>*Provide details below please* |
|---|---|---|---|---|---|
| | | | | | |

| 7. What motivated you to seek care at the clinic? Please tick all that apply to this visit | | | | | | | |
|---|---|---|---|---|---|---|---|
| Felt very ill | Family said I should come | Friends said I should come | Nurse or doctor said I should come | Someone else said I should come | Came to clinic for other reason | Worried that I might have TB | Other *Provide details below please* |
| | | | | | | | |

## SECTION 2 – TUBERCULOSIS DIAGNOSIS AND TREATMENT

| | Yes | No |
|---|---|---|
| 1. Had you ever been tested for TB before? | | |

| 2. Think about when you were tested for TB and please answer the following questions: | Yes | No |
|---|---|---|
| Was it a positive experience? | | |
| Did you have to wait long? | | |
| Was the nurse friendly? | | |
| Did she explain to you that she is testing you for TB? | | |
| Did she explain to you why she is testing you for TB? | | |
| Did she ask you to come back? | | |
| Did she ask you to come back on a specific day? | | |
| Did you go back to the clinic? | | |

| | Yes | No |
|---|---|---|
| 3. Did you go back to the clinic the first time you were tested to collect your test results? | | |

4. If you did not go back to the clinic, why not? Please tick three reasons that apply and rank them in order of importance

| | Yes | No | Rank |
|---|---|---|---|
| I forgot to go back | | | |
| I am still planning to go back to clinic | | | |
| I did not feel ill | | | |
| I do not think I have TB | | | |
| I have been too busy | | | |
| I am afraid of getting my test results | | | |

| | | | |
|---|---|---|---|
| I don't like the clinic | | | |
| The nurses at the clinic are unfriendly | | | |
| I am worried about side effects of treatment | | | |
| I could not get time off work | | | |
| I went to the clinic, but they could not give me the results | | | |
| I don't have time for TB treatment | | | |
| I don't have the money for TB treatment | | | |
| I am ashamed | | | |
| There is no privacy at the clinic | | | |
| I did not know that I had to go back to the clinic | | | |
| I went elsewhere, for example traditional healer or private practice | | | |

5. How easy is it for you to come to the clinic?

| 1. Very easy | 2. Easy | 3. Neither easy nor difficult | 4. Not easy/difficult | 5. Very difficult |
|---|---|---|---|---|
| | | | | |

6. How well did you understand the TB treatment process?

| 1. Very well | 2. Well | 3. Understand some things | 4. Not well | 5. Not at all |
|---|---|---|---|---|
| | | | | |

7. Please describe the treatment process for TB:

8. Was there anything that you thought would be different about the treatment process before you started?

| SECTION 3 – DIAGNOSIS AND TREATMENT JOURNEYS |
| --- |
| |

1. Please tell us your story about finding out that you had TB

2. Please tell us why you came to the clinic when you were first tested for TB. Also remember to tell us when you returned to collect your tests results

3. Please tell us your story of being treated for TB

# Appendix 5

**EForm Name**: REC: Humanities New Application

**Page**: Page 9

**Section**: 12.2 Method of Data Collection

**Question**: Attach Here (if applicable)

**File Name**: Questionnaire_201600831.docx

## QUESTIONNAIRE ABOUT YOUR HEALTH

| | |
|---|---|
| **Your name:** | |
| **Your clinic folder number:** | |
| **The address where you now live:** | |
| **I hereby allow for my records at the health center to be accessed and used for this study (signature required):** | |

## TICK THE CORRECT BOX/BOXES

SECTION 1 – MAKING CHOICES

| | | | |
|---|---|---|---|
| 1. Would you prefer to receive R200 guaranteed *today* or R250 guaranteed in *one month from today* (calculate and provide date)? | Today | 1 Month | → If "R250 One month" skip next question |
| | | | |
| 2. Would you prefer to receive R200 guaranteed *today* or R300 guaranteed in *one month from today* (calculate and provide date)? | Today | 1 Month | |
| | | | |
| 3. Which guaranteed amount would you like to receive in *one month from today* (calculate and provide date) that will make you just as happy as R200 *today*? | | | |
| 4. Would you prefer to receive R200 guaranteed *six months from today* or R250 guaranteed in *seven months from today* (calculate and provide date)? | 6 Months | 7 Months | → If "R250 Seven months" skip next question |
| | | | |
| 5. Would you prefer to receive R200 guaranteed *six months from today* or R300 guaranteed in *seven months from today* (calculate and provide date)? | 6 Months | 7 Months | |
| | | | |
| 6. Which guaranteed amount would you like to receive in *seven months from today* (calculate and provide date) that will make you just as happy as R200 *six months from today*? | | | |

7. Imagine the following situation: you are buying groceries and the total comes to R100. While you are standing in line, you see that the store offers a R10 discount if you pay in cash so you decide to pay cash:

   You look in your wallet and see that you have the cash so you will get the R10 discount. How would you feel about getting the R10 discount?

| 1. Very Unhappy | 2. Unhappy | 3. Neutral | 4. Happy | 5. Very Happy |
|---|---|---|---|---|
| | | | | |

8. Imagine the following situation: you are buying groceries and the total comes to R100. While you are standing in line, you see that the store offers a R10 discount if you pay in cash so you decide to pay cash:

   You look in your wallet and see that you don't have the cash, so you will not get the discount. How would you feel using your bank card (debit card) and not getting the R10 discount?

| 1. Very Unhappy | 2. Unhappy | 3. Neutral | 4. Happy | 5. Very Happy |
|---|---|---|---|---|
| | | | | |

| 9. Imagine the following situation: you are buying groceries and the total comes to R100. While you are standing in line, you see that the store charges a R10 extra charge for paying with your card and you decide to pay cash:<br><br>You look in your wallet and see that you have cash so you will not have to pay the extra charge. How would you feel paying in cash and not paying the R10 extra charges? | | | | |
|---|---|---|---|---|
| 1. Very Unhappy | 2. Unhappy | 3. Neutral | 4. Happy | 5. Very Happy |
| | | | | |

| 10. Imagine the following situation: you are buying groceries and the total comes to R100. While you are standing in line, you see that the store charges a R10 extra charge for paying with your card and you decide to pay cash:<br><br>You look in your wallet and see that you don't have the cash, so you will be paying the extra charge. How would you feel about using your card and paying the R10 extra charges? | | | | |
|---|---|---|---|---|
| 1. Very Unhappy | 1. Very Unhappy | 1. Very Unhappy | 1. Very Unhappy | 1. Very Unhappy |
| | | | | |

## SECTION 2 – GENERAL HEALTH AND SYMPTOMS

| | Yes | No |
|---|---|---|
| 1. Are you currently feeling unwell? | | |

| | M | M | Y | Y | Y | Y |
|---|---|---|---|---|---|---|
| 2. If yes, when did you start to feel unwell? | | | | | | |

| 3. If you are feeling unwell, did you experience any of the symptom/s below? Please tick all that apply | | | | | | | |
|---|---|---|---|---|---|---|---|
| Persistent cough | Cough with blood | Fever | Night sweats | Chest pain | Generally not feeling well | Weight loss | Other |
| | | | | | | | |

| 4. What symptoms are bothering you the most? Please tick all that apply | | | | | | | |
|---|---|---|---|---|---|---|---|
| Persistent cough | Cough with blood | Fever | Night sweats | Chest pain | Generally not feeling well | Weight loss | Other |
| | | | | | | | |

| 5. What motivated you to seek care today? Please tick all that apply to this visit | | | | | | | |
|---|---|---|---|---|---|---|---|
| Felt very ill | Family said I should come | Friends said I should come | Nurse or doctor said I should come | Someone else said I should come | Came to clinic for other reason | Worried that I might have TB | Other *Provide details below please* |
| | | | | | | | |

| 6. In the last week, what have you been worried about? | Tick all that apply | Rank worries from 1 to 10 (1 = most worried; 8 = least worried) |
|---|---|---|
| Feeding my family | | |
| My health | | |
| Being able to get to/from work | | |
| Children in my household being able to go to school | | |
| The health of children in my household or other family members | | |
| Finding work/ keeping my job | | |
| Going hungry | | |
| My relationship with my spouse or other family members | | |

| 7. How important are health concerns to you? | | | | |
|---|---|---|---|---|
| 1. Very Important | 2. Somewhat important | 3. Neutral | 4. Not very important | 5. Not at all important |
| | | | | |

| 8. How important is it to you to see a nurse and get the pills you need if you are ill? | | | | |
|---|---|---|---|---|
| 1. Very Important | 2. Somewhat important | 3. Neutral | 4. Not very important | 5. Not at all important |
| | | | | |

## SECTION 3 – TUBERCULOSIS KNOWLEDGE AND BELIEFS

1. [Name] has been diagnosed with tuberculosis. Please indicate whether you agree or disagree with each of the statements below.

| | 1. Strongly agree | 2. Agree | 3. Neutral | 4. Disagree | 5. Strongly disagree |
|---|---|---|---|---|---|
| This will make it hard for [Name] to get a job | | | | | |
| I would be comfortable working with [Name] | | | | | |
| I would be comfortable having [Name] over for dinner | | | | | |

| | | | | | |
|---|---|---|---|---|---|
| It is likely that [Name}'s wife/husband will leave him/her | | | | | |

| 2. A child in [Name]'s house has been coughing and losing weight. Please indicate whether you agree or disagree with each of the statements below. | | | | | |
|---|---|---|---|---|---|
| | 1. Strongly agree | 2. Agree | 3. Neutral | 4. Disagree | 5. Strongly disagree |
| The child should go to the clinic | | | | | |
| The child will make others sick | | | | | |
| [Name] should wait and see if the symptoms get better before taking the child to the doctor/nurse | | | | | |

| 3. Please answer yes or no to the following statements | Yes | No |
|---|---|---|
| TB can be easily cured | | |
| I know a lot about TB and its treatment | | |
| All people with TB will develop HIV/AIDS | | |
| One only needs to worry about TB if you are coughing blood | | |
| Curing TB takes a long time | | |
| TB medication is expensive | | |
| I know someone who has had TB | | |
| I know someone who has died from TB | | |
| People who do not get treated for TB are irresponsible | | |
| If you have TB, people do not respect you | | |
| It is the people that do not get treatment for TB that make other people sick | | |
| I know someone who was cured of TB | | |
| If you had TB before, you cannot get it again | | |
| TB treatment has bad side effects | | |
| You cannot work and be on TB treatment | | |
| TB can be cured, but the clinic treatment is not effective | | |
| Often people go on TB treatment, but are not cured | | |
| I think I might have TB | | |

| 4. How likely do you think it is that you have TB? | | | | |
|---|---|---|---|---|
| 1. Very likely | 2. Somewhat likely | 3. Neutral | 4. Unlikely | 5. Extremely unlikely |
| | | | | |

| 5. If you close your eyes and imagine a typical TB patient, please tick all of the characteristics that apply to him/her. Is the person: | | | |
|---|---|---|---|
| Young | | Rich | |
| Clean | | Female | |
| Old | | Poor | |
| Dirty | | Male | |
| Smoking | | Thin | |
| Drinking | | HIV positive | |
| Unemployed | | Look and feel very sick | |

## SECTION 4 – TUBERCULOSIS DIAGNOSIS AND TREATMENT

| 1. How easy is it for you to come to the clinic? | | | | |
|---|---|---|---|---|
| 1. Very easy | 2. Easy | 3. Neither easy nor difficult | 4. Not easy/difficult | 5. Very difficult |
| | | | | |

| 2. How well do you understand the TB treatment process? | | | | |
|---|---|---|---|---|
| 1. Very well | 2. Well | 3. Understand some things | 4. Not well | 5. Not at all |
| | | | | |

| 3. If you have TB, you will need to (select those that you agree with): | | | |
|---|---|---|---|
| Attend the clinic every day for 6 months | | Attend the clinic every day 12 months | |
| Attend the clinic every day for 24 months | | Receive injections every day for 2 months | |
| Take treatment for 6 months at a place that is convenient for me | | Not take medication, because none is available | |

| | Yes | No |
|---|---|---|
| 4. Do you intend to return to the clinic to collect your results? | | |
| 5. When will you return to the clinic to collect your results (DD/MM/YYYY)? | | |

## SECTION 5 – SOCIO-ECONOMIC AND LIFESTYLE

1. What type of house do you live in?

| | |
|---|---|
| Brick house | 1 |
| Traditional dwelling/hut/structure | 2 |
| Informal dwelling or shack | 3 |
| Other | -8 |

| 2. In your household is there: | Yes | No |
|---|---|---|
| Someone with a university degree | | |
| Someone with a job | | |
| Electricity | | |
| Hot water running from a tap | | |
| Television | | |
| Refrigerator/freezer | | |
| Satellite dish | | |
| Car | | |
| Someone with a mobile phone | | |

| | Yes | No |
|---|---|---|
| 3. Do you use electricity for heating? | | |
| 4. Do you use electricity for cooking? | | |

| | Yes | No | Unknown |
|---|---|---|---|
| 5. During the last 4 weeks, have you taken any drinks containing alcohol? | 1 | 0 | -5 |
| 6. During the last year have you ever drunk so much that you were unable to remember what you were doing? | 1 | 0 | -5 |

| | | |
|---|---|---|
| 7. Please tell us about your smoking habits: | Daily smoker | 1 |
| | Occasional smoker | 2 |
| | Ex-smoker | 3 |
| | Never smoked | 4 |

If smoker, occasional smoker or ex-smoker continue, if never-smoker skip to next section

| | | | |
|---|---|---|---|
| 8. How long have you/did you smoke for (Years) [999 if unknown] | | | |
| 9. How many cigarettes did/do you smoke per day? [999 if unknown] | | | |

| | Yes | No | Unknown |
|---|---|---|---|
| 10. Do you ever smoke marijuana/dagga? | | | |
| 11. Do you ever take other drugs such as cocaine, TIK or speed? | | | |

| PARTICIPANT FOLLOW-UP & CONTACT DETAILS | | |
|---|---|---|
| **Are willing to receive sms information from the study team?** | YES☐ | NO☐ |
| **Are willing to be contacted at a later stage to answer more questions?** | YES☐ | NO☐ |
| **If you are willing to receive a sms or to be contacted at a later stage to answer more questions** | | |
| Please provide your telephone or cell number: | | |
| Please provide the name and telephone or cell number of a family member who we can contact you through: | | |
| Please provide the name or telephone or cell number of a friend who we can contact you through: | | |

| TO BE COMPLETED BY FIELD RESEARCHER | |
|---|---|
| Date of Interview | ____/______/______ (DD/MM/YYYY) |
| Researcher's Name | |
| Start Time - End time | ______________ to _____________ (eg 11:30 to 12:15) |
| Laboratory test results | Gene Xperrt Pos☐ Neg☐ Date received____/______/______ (DD/MM/YYYY) |
| | Smear result Pos☐ Neg☐ Date received____/______/______ (DD/MM/YYYY) |
| | Culture result Pos☐ Neg☐ Date received____/______/______ (DD/MM/YYYY) |
| Date patient first returned to facility | ____/______/______ (DD/MM/YYYY) Result provided YES☐ NO☐ |
| Reason if result not provided | |

# Appendix 6

**EForm Name**: REC: Humanities New Application

**Page**: Page 10

**Section**: 15. Additional documents to be uploaded (Please click on the yellow "+" in the right hand corner to add)

**Question**: Upload

**File Name**: cover_letter20160905.pdf

UNIVERSITEIT•STELLENBOSCH•UNIVERSITY
jou kennisvennoot • your knowledge partner

Dear Dr Lyn Horn,

**Re: Social and psychological/behavioural factors influencing non-initiation of TB treatment**

We hereby submit an ethics application for a new pilot study that aims to investigate the causes of non-initiation of tuberculosis treatment in Cape Town, Western Cape.

The study is undertaken as a multi-disciplinary collaboration (economics/clinical), the innovative and dominant elements in the study is motivated by economic theory and specifically, a new hybrid field in Economics drawing on psychology and economics (behavioural economics). We therefore feel strongly that this study is best assessed and supported by social scientists, particularly given that the ethical board at Stellenbosch University includes a psychologist and an industrial psychologist.

Please also note that this is a small exploratory pilot study funded via seed grants from the World Bank and the Gates Foundation.

One further consideration is timelines. I know that you are bound by the formal cycles of your process but would appreciate any prioritisation or fast tracking that is possible. We need to be in the field in January and still need to obtain approval for accessing City of Cape Town sites. Although exploratory, this is a high stakes project for us because it includes two of the top ten health economists (Adam Wagstaff and Eddy van Doorslaer, rated according to recent bibliometric study) and a number of important other stakeholders including our funders as well as our institutional partners City of Cape Town and ECDOH. It is therefore crucial for us that we do all that we can to ensure that we can be in the field in January 2017.

Yours sincerely,

Rochelle Jacobs

# Appendix 7

**EForm Name**: REC: Humanities New Application

**Page**: Page 10

**Section**: 15. Additional documents to be uploaded (Please click on the yellow "+" in the right hand corner to add)

**Question**: Upload

**File Name**: REC_amend_letter.docx

UNIVERSITEIT•STELLENBOSCH•UNIVERSITY
jou kennisvennoot • your knowledge partner

Dear Dr Lyn Horn,

**Re: Response to Modifications REC Humanities for Proposal SU-HSD-003565**

Please find below comments and responses to the requested modifications on the ethical clearance proposal SU-HSD-003565.

1. PARTICIPATION SELECTION AND RECRUITMENT:
   Permission will be attained from the Western Cape Department of Health in order to obtain permission to contact patients who have completed their TB treatment journeys. Such an application can only be undergone once ethical clearance has been obtained from Stellenbosch University.

2. PROTECTION OF PARTICIPANTS PRIVACY AND CONFIDENTIALITY
   Participants' privacy is ensured through the use of tablets in conducting the survey. Should further privacy measures be necessary, we will endeavour to make them available.

3. INFORMED CONSENT AND ASSENT PROCESS AND FORM
   The ICFs for this study have been entirely redone and are attached in their new form. They are also attached as amendments to Annexure A of the original application (now Annexure A1 and A2 to differentiate between the two forms) and these changes have been tracked as requested.

3.1 The informed consent form (ICF) has been amended to state that interviewers will wear masks for their own protection. This amendment is included on pages 14 and 18 of Annexure A1 and A2 respectively.

3.2 The two ICF's attached in Section 9 of the REC application have been amended in order to ensure completeness and to reflect the suggested changes, as they appear in Annexure A1 and A2.

3.3 Annexures A1 and A2 have been amended to state that: This study has been approved by the Research Ethics Committee: Humanities… Such changes are seen on pages 14 and 18 respectively.

3.4 Ms. Maléne Fouché's details have been added to the ICF's, as requested. These details appear on pages 15 and 19 of Annexure A1 and A2 respectively.

3.5 Each ICF has been amended to include an overview of the nature of the study and questions asked. Such descriptions appear in the ICFs as separately attached as well as in Annexure A1 and A2 on pages 14-15 and 18-19 respectively.

3.6 The ICF's and questionnaires will be translated into isiXhosa and Afrikaans to accommodate those patients who do not speak English. These translated versions will be field tested to ensure that they are equivalent to the English one. Such translations can only occur once the instrument itself has been field tested.

3.7 We appreciate your decision about waiving consent for the identification of TB patients for our patient journey interviews. We will make it explicit on the ICF that patients can all still opt out. Such information is contained in Annexure A1 and A2 on pages 15 and 19 of Annexure A1 and A2 respectively.

3.8 The SU ICF has been used as a template for expanding the ICF presented in this proposal. All amendments described above have been incorporated into this template. In the ICFs submitted here, pages 1 to 2 will be given to the participants to retain as they contain the informational element of the ICF.

4. ADEQUATE MITIGATION OF RISK/COUNSELLING SERVICES

The contact details for the HIV/TB counsellor at relevant clinics will be added to the ICF, once city approval has been given so that we ensure the most up-to-date details are provided. In the interim, space has been made on the ICF for this information and explicit reference to the availability of such counsellors has been made. Field workers may also be instructed to emphasize their availability to patients. The contact details of counsellors will appear on page 2 of each ICF, which is given to the participant to retain.

5. INSTITUTIONAL AND EXTERNAL PERMISSIONS

Sorry, this is a mistake based on the previous application where we had positioned this as auxiliary survey to an existing large TB research study. As mentioned in point 1, we still need to seek formal permission from the City of Cape Town Health and will only be able to do so after we have obtained ethical clearance from the university.

6. ADDITIONAL COMMENTS

6.1 The title of the project has been amended for consistency. The correct title is "Social and psychological/behavioural factors influencing non-initiation of TB treatment".

6.2 Field worker insurance shall be obtained.

Rochelle Jacobs
