# Supplementary material for: SMS nudges as a tool to reduce tuberculosis treatment delay and pretreatment loss to follow-up. A randomized controlled trial
Source: PLoS One. 2019 Jun 20;14(6):e0218527. doi: 10.1371/journal.pone.0218527 (PMC6586322; doi:10.1371/journal.pone.0218527)
Supplement: S5 File — (DOCX) [file pone.0218527.s005.docx]

S5 Table Distribution of sample across three study clinics

| Clinic | Control | SMS1 | SMS2 | Total |
| --- | --- | --- | --- | --- |
|  |  |  |  |  |
| Clinic A | 47 | 89 | 85 | 221 |
| Clinic B | 18 | 37 | 34 | 89 |
| Clinic C | 36 | 78 | 82 | 196 |
|  |  |  |  |  |
| Total | 101 | 204 | 201 | 506 |
