# Supplementary material for: SMS nudges as a tool to reduce tuberculosis treatment delay and pretreatment loss to follow-up. A randomized controlled trial
Source: PLoS One. 2019 Jun 20;14(6):e0218527. doi: 10.1371/journal.pone.0218527 (PMC6586322; doi:10.1371/journal.pone.0218527)
Supplement: S6 File — (DOCX) [file pone.0218527.s006.docx]

S6 Table Baseline characteristics (per-protocol sample)

|  | Control (n=97) | | SMS (n=318) | | SMS1 (n=163) | | SMS2 (n=155) | |
| --- | --- | --- | --- | --- | --- | --- | --- | --- |
| Variable | N/mean | %/SD | N/mean | %/SD | N/mean | %/SD | N/mean | %/SD |
| Female | 42 | 43.3 | 148 | 46.5 | 70 | 42.9 | 78 | 50.3 |
| College educated | 12 | 12.4 | 45 | 14.2 | 26 | 16.0 | 19 | 12.3 |
| Employed | 59 | 60.8 | 199 | 62.6 | 103 | 63.2 | 96 | 61.9 |
| Electricity | 92 | 94.8 | 295 | 92.8 | 149 | 91.4 | 146 | 94.2 |
| Running water | 12 | 12.4 | 22 | 6.9 | 13 | 8.0 | 9 | 5.8 |
| TV | 85 | 87.6 | 287 | 90.3 | 149 | 91.4 | 138 | 89.0 |
| Fridge | 81 | 83.5 | 272 | 85.5 | 136 | 83.4 | 136 | 87.7 |
| Satellite | 36 | 37.1 | 96 | 30.2 | 58 | 35.6 | 38 | 24.5 |
| Car | 18 | 18.6 | 45 | 14.2 | 25 | 15.3 | 20 | 12.9 |
| Mobile phone in household | 88 | 90.7 | 278 | 87.4 | 142 | 87.1 | 136 | 87.7 |
| Hungry | 26 | 26.8 | 94 | 29.6 | 48 | 29.4 | 46 | 29.7 |
| Age | 39.92 | 13.01 | 39.76 | 11.73 | 38.96 | 11.52 | 40.59 | 11.92 |
