# Supplementary material for: SMS nudges as a tool to reduce tuberculosis treatment delay and pretreatment loss to follow-up. A randomized controlled trial
Source: PLoS One. 2019 Jun 20;14(6):e0218527. doi: 10.1371/journal.pone.0218527 (PMC6586322; doi:10.1371/journal.pone.0218527)
Supplement: S7 File — (DOCX) [file pone.0218527.s007.docx]

**S7 Table** Results for subsample not known to be HIV-positive

|  | N | | Controls | Any SMS | | p | | SMS1 | | SMS2 | | p1 | | p2 | | p3 | |  |
| --- | --- | --- | --- | --- | --- | --- | --- | --- | --- | --- | --- | --- | --- | --- | --- | --- | --- | --- |
| Returned (as % cases analyzed | | | |  | |  | |  | |  | |  | |  | |  | |  |
| ITT | 322 | 53/66 (80.3%) | | | 207/256 (80.9%) | | 0.919 | | 102/125 (81.6%) | | 105/131 (80.2%) | | 0.827 | | 0.980 | | 0.769 | |
| PP | 268 | 50/62 (80.6%) | | | 167/206 (81.1%) | | 0.941 | | 84/103 (81.6%) | | 83/103 (80.6%) | | 0.885 | | 0.992 | | 0.859 | |
| Time-to-return (mean days) | | | |  | |  | |  | |  | |  | |  | |  | |  |
| ITT | 322 | 11.1 | | | 10.7 | | 0.864 | | 11.0 | | 10.4 | | 0.967 | | 0.788 | | 0.786 | |
| PP | 268 | 10.9 | | | 10.8 | | 0.970 | | 11.0 | | 10.5 | | 0.952 | | 0.896 | | 0.825 | |
| Returned within 2 business days (as % recruits) | | | | | |  | |  | |  | |  | |  | |  | |  |
| ITT | 322 | 42/66 (63.6%) | | | 154/256 (60.2%) | | 0.605 | | 75/125 (60%) | | 79/131 (60.3%) | | 0.624 | | 0.650 | | 0.960 | |
| PP | 268 | 40/62 (64.5%) | | | 128/206 (62.1%) | | 0.734 | | 65/103 (63.1%) | | 63/103 (61.2%) | | 0.855 | | 0.667 | | 0.774 | |
| Returned within 5 business days (as % recruits) | | | | | |  | |  | |  | |  | |  | |  | |  |
| ITT | 322 | 51/66 (77.3%) | | | 193/256 (75.4%) | | 0.750 | | 96/125 (76.8%) | | 97/131 (74%) | | 0.941 | | 0.621 | | 0.609 | |
| PP | 268 | 49/62 (79%) | | | 156/206 (75.7%) | | 0.591 | | 80/103 (77.7%) | | 76/103 (73.8%) | | 0.837 | | 0.446 | | 0.516 | |
| Returned within 10 business days (as % recruits) | | | | | |  | |  | |  | |  | |  | |  | |  |
| ITT | 322 | 51/66 (77.3%) | | | 203/256 (79.3%) | | 0.719 | | 99/125 (79.2%) | | 104/131 (79.4%) | | 0.758 | | 0.732 | | 0.970 | |
| PP | 268 | 49/62 (79%) | | | 164/206 (79.6%) | | 0.921 | | 82/103 (79.6%) | | 82/103 (79.6%) | | 0.929 | | 0.929 | | 1.000 | |
| Returned within 20 business days (as % recruits) | | | | | |  | |  | |  | |  | |  | |  | |  |
| ITT | 322 | 51/66 (77.3%) | | | 207/256 (81.2%) | | 0.477 | | 101/125 (80.8%) | | 106/131 (81.5%) | | 0.565 | | 0.480 | | 0.880 | |
| PP | 268 | 49/62 (79%) | | | 167/206 (81.1%) | | 0.722 | | 83/103 (80.6%) | | 84/103 (81.6%) | | 0.809 | | 0.692 | | 0.859 | |

Notes: p for returned (as % recruits) and returned within *x* business days (as % recruits) is Pearson’s χ^2^ test of equal proportions (of returners / returners-within-*x*-days) across intervention and control groups. p for time-to-return (mean days) is a test of equality of means. p_1_ is test for SMS1 vs. control, p_2_ is test for SMS2 vs. control, p_3_ is test of equality across the two SMS groups.
