# Supplementary material for: SMS nudges as a tool to reduce tuberculosis treatment delay and pretreatment loss to follow-up. A randomized controlled trial
Source: PLoS One. 2019 Jun 20;14(6):e0218527. doi: 10.1371/journal.pone.0218527 (PMC6586322; doi:10.1371/journal.pone.0218527)
Supplement: S8 File — (DOCX) [file pone.0218527.s008.docx]

**S8 Fig** SMS1 and SMS2 results (per protocol)

Notes: lines represent 95% confidence intervals.
